# Payment Source Shift for Surgical Care Among Veterans Enrolled in Medicare Advantage Plans

**DOI:** 10.1001/jamahealthforum.2025.0827

**Published:** 2025-06-09

**Authors:** Winta T. Mehtsun, Yanlei Ma, Ellen Latsko, Jie Zheng, Jessica Phelan, E. John Orav, Thomas C. Tsai, Austin B. Frakt, Steven D. Pizer, Melissa M. Garrido, Jose F. Figueroa

**Affiliations:** 1 Department of Surgery, University of California, San Diego; 2 Department of Health Policy & Management, Harvard T.H. Chan School of Public Health, Boston, Massachusetts; 3 Division of General Internal Medicine and Primary Care, Brigham and Women’s Hospital, Boston, Massachusetts; 4 Department of Surgery, Brigham and Women’s Hospital, Boston, Massachusetts; 5 VA Boston Healthcare System, Boston, Massachusetts; 6 Boston University School of Public Health, Boston, Massachusetts

## Abstract

**Importance:**

There is growing concern that Medicare Advantage (MA) plans are shifting the costs of care to the Veterans Health Administration (VHA) for veterans dually enrolled in both systems, particularly in high-veteran MA plans that disproportionately enroll veterans. However, empirical evidence evaluating the sources of payment for veterans' surgical care is lacking.

**Objective:**

To evaluate differences in payment sources for surgical care between high-veteran MA plans and other MA plans.

**Design, Setting, and Participants:**

This cross-sectional study used 2021 US national MA and VHA data from veterans dually enrolled in MA and VHA care for inpatient surgical episodes at VHA facilities (VHA-paid direct care), non-VHA community hospitals paid by VHA (VHA-paid community care), and community hospitals paid by MA (MA-paid community care) among veterans dually enrolled in MA and VHA care. Data were analyzed from April 1, 2024, to November 30, 2024.

**Exposure:**

Enrollment in high-veteran MA plans.

**Main Outcomes and Measures:**

Likelihood of utilizing VHA-direct care, VHA-paid community care, and MA-paid community care. High-veteran MA plans were defined as plans with 20% or more veteran enrollees; others were categorized as other MA plans. Multinomial logistic regression was used to evaluate the association of veteran enrollment in high-veteran MA plans with the likelihood of surgical care paid by each payment source, adjusting for veteran and surgery characteristics, and state fixed effects. Stratified analyses were conducted based on surgical complexity and source of admission.

**Results:**

A total of 54 754 inpatient surgical episodes were analyzed, including 53 036 male (96.9%); 3133 Hispanic (5.7%), 47344 non-Hispanic Black (13.4%), 2933 non-Hispanic White (78.4%), and 1354 other or unknown race and ethnicity (2.5%); 601 (1.1%) were younger than 55 years, 3301(6.0%) aged 55 to 64 years, 22 381 (40.9%) aged 65 to 74 years, and 28471 (52%) aged 75 or older. Among these episodes, 52.1% were through MA-paid community care, 18.8% through VHA-direct care, and 29.1% through VHA-paid community care. Veteran enrollees in high-veteran MA plans were significantly less likely to have MA-paid surgeries (adjusted difference, −25.7 percentage points; 95% CI, −26.7 to 24.6 percentage points) and more likely to have surgeries paid through VHA-direct care (adjusted difference, 11.0 percentage points; 95% CI, 10.0-12.0 percentage points) and VHA-paid community care (adjusted difference, 14.7 percentage points; 95% CI, 13.6-15.8 percentage points) compared with veterans in other MA plans. As surgical complexity increased, differences in the use of VHA-paid direct care narrowed between high-veteran MA and other MA plans. Payment source differences were also less pronounced for nonelective surgeries admitted through emergency departments.

**Conclusions and Relevance:**

The findings of this cross-sectional study suggest substantial cost shifting in veterans’ surgical care from MA to VHA among high-veteran MA plans, underscoring the urgent need for policy reforms to improve the efficiency of veterans’ care.

## Key Points

**Question:**

Are high-veteran Medicare Advantage (MA) plans more likely than other MA plans to shift payments for inpatient surgical care to the Veterans Health Administration (VHA)?

**Findings:**

In this cross-sectional study including 54 754 inpatient surgical episodes by VHA enrollees enrolled in MA plans in 2021, VHA enrollees enrolled in high-veteran MA plans were significantly less likely to have surgeries paid by MA and instead more likely to receive surgical care paid by the VHA compared with other MA plans with lower veteran enrollment.

**Meaning:**

These findings indicate substantial payment shifting from MA to VHA among high-veteran MA plans, underscoring the need for policy reforms to ensure more efficient allocation of federal resources when caring for veterans.

## Teaser

This cross-sectional study evaluates differences in payment sources for surgical care between high-veteran Medicare Advantage plans and other Medicare Advantage plans.

## Introduction

An increasing number of Medicare-eligible veterans are enrolling in Medicare Advantage (MA) over traditional Medicare (TM).^1,2,3,4^ In 2022, nearly 1.3 million veterans enrolled in MA plans, accounting for approximately 34% of the Medicare-enrolled veteran population.^5^ For veterans dually covered by the Veterans Health Administration (VHA) and MA plans, there are 3 primary payment sources if they require inpatient hospital care: (1) direct care through VHA inpatient hospitals (VHA-direct care), (2) VHA-paid care in non-VHA hospitals commonly referred to as community hospitals (VHA-paid community care), and (3) MA-paid care provided in non-VHA community hospitals (MA-paid community care) similar to nonveteran MA enrollees.

Amid ongoing MA growth, there are questions about increases in duplicative wasteful federal spending for the care of veterans.^1,2,4,6,7,8,9,10^ This is because the Centers for Medicare & Medicaid Services (CMS) pays MA plans fixed capitated per-member-per-month rates for comprehensive care regardless of the actual volume of services, while the VHA does not bill MA plans for Medicare-covered services provided to MA-enrolled veterans.^11^ Between 2011 and 2020, the VHA paid more than $78 billion for veterans enrolled in MA despite MA receiving full payments from CMS.^2^ Such duplicative payments for MA-enrolled veterans are even more notable with the passage of the VHA Maintaining Internal Systems and Strengthening Integrated Outside Networks (MISSION) Act of 2018,^12^ which substantially expanded veterans’ access to VHA-paid community care.^13,14^

More recently, MA plans have emerged specifically marketing to and enrolling veterans known as high-veteran MA plans.^4,15,16,17,18^ From 2016 to 2022, the number of high-veteran MA plans almost doubled; by 2022, these plans enrolled nearly 1 in 5 MA-enrolled veterans.^4^ Compared with other MA plans, veterans enrolled in high-veteran MA plans were significantly less likely to submit a single Medicare service claim,^4^ raising questions about whether these plans may be shifting the cost of care to the VHA system while keeping the CMS fixed per-member-per-month payments that would otherwise have been spent on veterans’ care.^18,19^ Additionally, there is no clear evidence that these plans offer tangible health care benefits for veterans.

Given the high costs associated with surgical care, which is projected to constitute more than half of Medicare expenditures in 2025,^20,21^ inpatient surgical care may be particularly susceptible to duplicative spending among MA-enrolled veterans. However, empirical evidence evaluating the sources of payment for veterans’ surgical care in MA plans is lacking. Existing research has primarily focused on comparing the quality and outcomes of veteran surgical care across different settings^22,23^ or examining the average cost to the VHA for specific procedures.^23,24^ Therefore, data on the source of payment for surgeries among veterans dually enrolled in MA may help inform federal policy efforts to reduce waste and streamline health care spending for the older veteran population.

This study had 3 objectives. First, we evaluated the characteristics of MA-enrolled veterans receiving surgical care through MA-paid community care, VHA-paid community care, and VHA-paid direct care. Second, we assessed differences in payment sources for inpatient surgical episodes between veterans enrolled in high-veteran MA plans vs other MA plans. Third, we examined whether these differences vary by surgical complexity or source of admission.

## Methods

### Data

This evaluation was for operational VHA purposes and considered nonresearch by the VHA Boston Healthcare System’s Research and Development Committee and thus institutional review board oversight was waived. The reporting of results in this study followed the Strengthening the Strengthening the Reporting of Observational Studies in Epidemiology (STROBE) reporting guideline. We used the VHA Planning Systems Support Group Geocoded Enrollee File^25^ and the Medicare Beneficiary Summary File^26^ to identify veterans who were dually enrolled in MA plans and VHA care in 2021. These data also provided veterans’ demographic information and priority group designation, which determines veterans’ cost-sharing levels for VHA care^27^ (eTable 1 in Supplement 1).

The VHA Corporate Data Warehouse was used to identify surgical care delivered by VHA physicians and facilities (ie, VHA-paid direct care). The Consolidated Data Set of the VHA Office of Integrated Veteran Care was used to identify VHA-paid inpatient surgical care from non-VHA physicians and facilities (ie, VHA-paid community care). The 100% MA encounter data were used to identify MA-paid inpatient surgical care. Inpatient surgical care episodes were identified using Diagnosis Related Group (DRG) codes^28^ (eTable 2 in Supplement 1).

Our sample included veterans who were both continuously enrolled in MA plans and eligible for VHA care throughout 2021. Veterans not enrolled in VHA care, VHA enrollees without surgical episodes, and those enrolled in MA plans for less than 12 months were excluded. For VHA enrollees who incurred multiple inpatient surgical episodes in 2021, to avoid correlation, we randomly selected one episode per VHA enrollee for analyses. Data were analyzed from April 1, 2024, to November 30, 2024.

### Identification of High-Veteran MA Plans

We defined a high-veteran MA plan as an MA plan with VHA enrollees accounting for 20% or more of their enrollment, corresponding to approximately the 95th percentile of VHA enrollee enrollment among all MA plans.^4^ We selected 20% as the threshold because no MA plan has a plan service area with VHA enrollees exceeding 20% of its total Medicare population (eFigure 1 in Supplement 1). The remaining MA plans were designated as other MA plans. We excluded cost plans, Medical Savings Account plans, plans with fewer than 100 beneficiaries, and those without any VHA enrollees.

### Statistical Analysis

We conducted 3 sets of analyses. First, we compared the characteristics of VHA enrollees receiving inpatient surgical care through each of the 3 payment sources. We used χ^2^ tests to assess differences in the proportions of characteristics among VHA enrollees receiving surgical care through these payment sources.

Second, we compared the likelihood of VHA enrollees utilizing each payment source between high-veteran MA plans and other MA plans. We examined the unadjusted proportions of inpatient surgical care paid through each payment source for high-veteran MA plans and other MA plans. We then estimated a multinomial logistic regression model to evaluate the association between veteran enrollment in high-veteran MA plans and the likelihood of their surgical care being paid by each of the 3 payment sources. The dependent variable is the payment source of the surgical episode, and the key explanatory variable is whether the VHA enrollee is enrolled in a high-veteran MA plan. Additional explanatory variables include VHA enrollees’ age group (younger than 55, 55-64, 65-74, and 75 years or older), sex, race and ethnicity (non-Hispanic White, non-Hispanic Black, Hispanic, other), dual-eligibility for Medicaid (not eligible, eligible for full Medicaid benefits, eligible for partial Medicaid benefits), rurality (urban, rural, highly rural), and priority group designations (priority group 1 through 8). Race and ethnicity were defined using the Research Triangle Institute Race Code variable and were analyzed given the known racial and ethnic differences in care utilization patterns among veterans.^1^ Other race and ethnicity includes American Indian or Alaska Native, Asian or Other Pacific Islander, as well as any other race and ethnicity categories that are not Black, Hispanic, or non-Hispanic White. Models included state fixed-effects and DRG weight quintiles to account for variation in care capacity and surgical complexity.^28^ We reported adjusted probabilities for surgical care paid through each payment source for high-veteran MA plans and other MA plans, as well as adjusted probability differences between the two types of plans.

Third, we stratified analyses by surgical complexity using DRG weight quintiles, which reflect the average resource intensity required to care for cases in that particular DRG relative to the average resources used to treat cases in all DRGs.^28^ Base DRG weights were used since community hospitals may be incentivized to more aggressively capture risk through DRG modifiers while the VHA hospitals do not necessarily do so.^29^ We also stratified analyses by the source of admission, classifying surgical episodes as nonelective if the VHA enrollee was admitted through the emergency department and as elective otherwise. We estimated separate multinomial logistic regression models for each DRG quintile and source of admission to assess the association between veteran enrollment in high-veteran MA plans and the adjusted probability of their surgical care being paid by each of the 3 payment sources, adjusting for the same set of covariates.

In sensitivity analyses, to understand the extent of cost shifting between Medicare and VHA by high-veteran MA plans, we estimated a 2-part logistic regression model instead of a multinomial logistic regression model to assess the association between veteran enrollment in high-veteran MA plans and the payment source of their surgical care. Additionally, given known issues of data incompleteness of MA encounter data, we repeated our analyses including only MA plans that are deemed as having high data completeness.^30^

Analyses were performed using SAS version 9.4 (SAS Institute Inc) and Stata version 18 (StataCorp LLC). The threshold for statistical significance was *P* < .05 using 2-sided tests.

## Results

The sample consisted of 54 754 inpatient surgical episodes by VHA enrollees enrolled in MA in 2021 (Table 1), of which 28 544 (52.1%) were paid by MA plans, 10 275 (18.8%) were provided through VHA-paid direct care, and 15 935 (29.1%) were provided through VHA-paid community care (Table 1). Compared with VHA enrollees whose surgical episodes were paid by MA plans, those incurring surgeries through VHA-paid care (including both VHA-paid direct care and VHA-paid community care) were younger and more likely to belong to the highest priority group (17.4% for MA-paid community care vs 36.3% for VHA-paid community care and 33.3% for VHA-paid direct care) (Table 1). In addition, VHA enrollees who incurred surgery through VHA-paid direct care were more likely to be non-Hispanic Black (20.4% for VHA-paid direct care vs 12.0% for MA-paid community care and 11.4% for VHA-paid community care), and those who incurred surgery through VHA-paid community care were more likely to reside in rural areas (44.8% for VHA-paid community care vs 30.7% for MA-paid community care and 30.1% for VHA-paid direct care) (Table 1).

**Table 1.** Characteristics of VHA Enrollees Receiving Inpatient Surgical Care in 2021^a^

Characteristic	Enrollees, No. (%)		
	MA-paid community care (n = 28 544)	VHA-paid community care (n = 15 935)	VHA-paid direct care (n = 10 275)
Age group, y			
<55	252 (0.9)	205 (1.3)	144 (1.4)
55-64	1281 (4.5)	1159 (7.3)	861 (8.4)
65-74	9779 (34.3)	7378 (46.3)	5224 (50.8)
≥75	17 232 (60.4)	7193 (45.1)	4046 (39.4)
Sex			
Female	737 (2.6)	593 (3.7)	388 (3.8)
Male	27 807 (97.4)	15 342 (96.3)	9887 (96.2)
Race and ethnicity^b^			
Hispanic	1571 (5.5)	788 (5.0)	774 (7.5)
Non-Hispanic Black	3423 (12.0)	1814 (11.4)	2097 (20.4)
Non-Hispanic White	22 868 (80.1)	12 901 (81.0)	7164 (69.7)
Other/unknown^c^	682 (2.4)	432 (2.7)	240 (2.3)
Dual eligibility for Medicaid benefits	3711 (13.0)	1756 (11.0)	1363 (13.3)
Original reason for Medicare			
Old-Age, Survivors, and Disability Insurance program	21 365 (74.9)	10 176 (63.9)	6523 (63.5)
DIB	7011 (24.6)	5694 (35.7)	3668 (35.7)
ESKD	83 (0.3)	29 (0.2)	34 (0.3)
DIB and ESKD	85 (0.3)	36 (0.2)	50 (0.5)
Region			
Northeast	5089 (17.8)	1925 (12.1)	1407 (13.7)
Midwest	6557 (23.0)	4352 (27.3)	2544 (24.8)
South	11 467 (40.2)	7089 (44.5)	4225 (41.1)
West	5098 (17.9)	2511 (15.8)	1855 (18.1)
US territories	333 (1.2)	58 (0.4)	244 (2.4)
Rural^d^	8766 (30.7)	7140 (44.8)	3090 (30.1)
Priority group^e^			
1	4955 (17.4)	5791 (36.3)	3425 (33.3)
2	1576 (5.5)	1041 (6.5)	701 (6.8)
3	3516 (12.3)	2066 (13.0)	1370 (13.3)
4	942 (3.3)	664 (4.2)	657 (6.4)
5	6280 (22.0)	3678 (23.1)	2461 (24.0)
6	1781 (6.2)	416 (2.6)	318 (3.1)
7A	53 (0.2)	20 (0.1)	20 (0.2)
7C	2207 (7.7)	409 (2.6)	350 (3.4)
8A-8D	7234 (25.3)	1850 (11.6)	973 (9.5)

Abbreviations: DIB, disability insurance benefits; ESKD, end-stage kidney disease; MA, Medicare Advantage; VHA, Veterans Health Administration.

^a^ Differences among 3 payment sources were statistically significant at *P* < .001 for each demographic group. Analyses limited to VHA enrollees who were continuously enrolled in MA plans in a given year. VHA enrollees residing outside 50 states and Washington, DC, were excluded from the analyses.

^b^ Race and ethnicity classification was derived from the Research Triangle Institute Race Code in the Medicare Beneficiary Summary File.

^c^ Other race and ethnicity includes American Indian or Alaska Native, Asian or Other Pacific Islander, as well as any other race and ethnicity categories that are not Black, Hispanic, or non-Hispanic White.

^d^ Rurality designation was obtained from Veterans Health Administration Planning Systems Support Group Geocoded Enrollee Files.

^e^ Priority groups for veterans are established by the Veterans Health Administration to determine veterans’ eligibility and priority for various VHA benefits and services based on factors like service-connected disabilities, income levels, and military decorations. It determines how much (if any) a veteran will have to pay toward the cost of their care. eTable 1 in Supplement 1 presents detailed information for priority group designations.

### Payment Sources for High-Veteran MA Plans vs Other MA Plans

**Figure 1.**
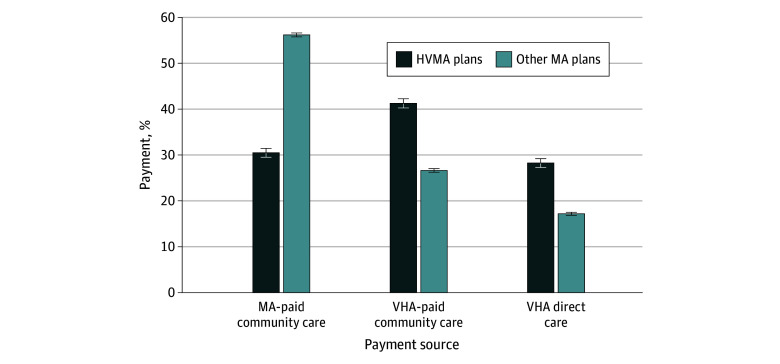
Adjusted Inpatient Surgery Payment Sources for Veterans Health Administration (VHA) Enrollees in High-Veteran MA Plans vs Other Medicare Advantage (MA) Plans, 2021 High-veteran MA plans are defined as MA plans that enroll VHA enrollees exceeding 20% of their total enrollment in 2021. Employer-direct private fee-for-service plans, cost plans, Medicare Medical Savings Account plans, and plans with fewer than 100 enrollees were excluded from the analyses. Adjusted share represents the proportion of surgical episodes that are covered by MA plans, VHA-paid community care, and VHA-paid direct care, estimated using a multinomial logistic regression model that regress payment source on enrollment in high-veteran MA plan, adjusting for VHA enrollees’ age, gender, race and ethnicity, Medicare and Medicaid dual eligibility, rurality, priority group designations, surgical complexity, and state fixed effects. HVMA indicates high-veteran Medicare Advantage. Whiskers indicate the 95% CIs of the estimated value.

**Figure 2.**
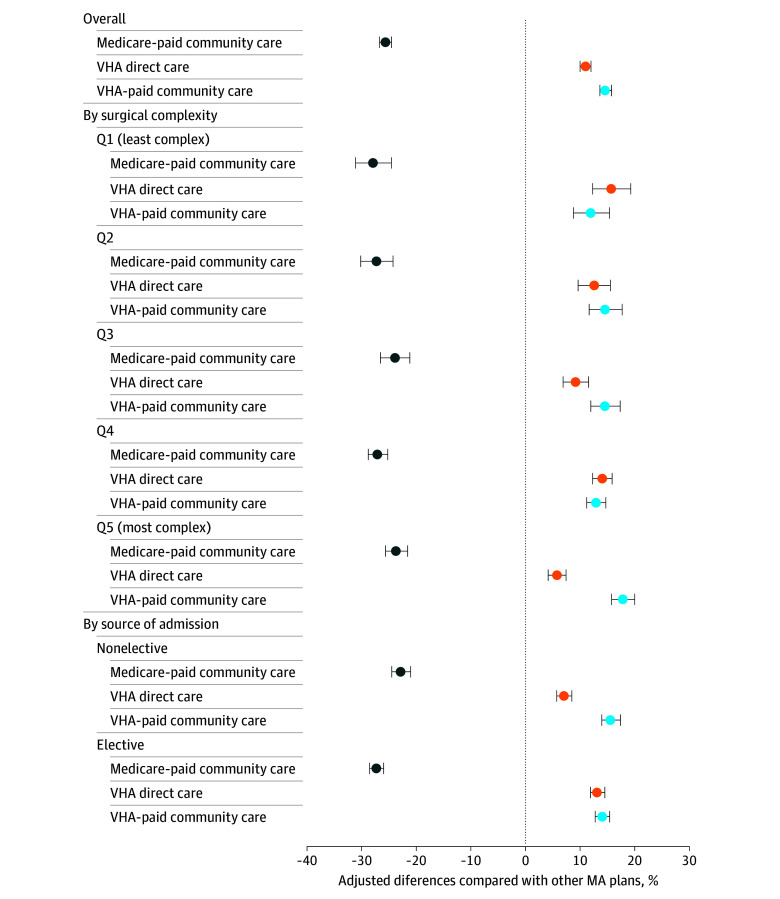
Adjusted Differences in Payment Sources for Inpatient Surgeries Between High-Veteran Medicare Advantage (MA) Plans vs Other MA Plans, 2021 High-veteran MA plans are defined as MA plans that enroll Veterans Health Affairs (VHA) enrollees exceeding 20% of their total enrollment in 2021. Cost plans, Medicare Medical Savings Account plans, and plans with fewer than 100 enrollees were excluded from the analyses. Multinomial logistic regression models were estimated to evaluate the association between veteran enrollment in high-veteran MA plans and the likelihood of their surgical care being paid by each of the 3 payment sources. Separate regression models were estimated for each surgical complexity quintile and each source of admission. The dependent variable is the payment source of the surgical episode, and the key explanatory variable is whether the VHA enrollee is enrolled in a high-veteran MA plan. Adjusted difference represents the difference in the proportion of surgical episodes that are covered by MA plans, VHA-paid community care, and VHA-paid direct care for VHA enrollees in high-veteran MA plans relative to other MA plans. Analyses adjusted for VHA enrollees’ age, gender, race and ethnicity, Medicare and Medicaid dual eligibility, rurality, priority group designations, surgical complexity, and state fixed effects. Q indicates quintile. Whiskers indicate the 95% CIs of the estimated value.

Of the surgical episodes incurred by VHA enrollees in high-veteran MA plans, 24.8% were paid by MA plans compared with 57.5% of episodes by VHA enrollees in other MA plans (eFigure 2 in Supplement 1). In adjusted analyses, 30.5% of inpatient surgical episodes for VHA enrollees in high-veteran MA plans were paid by MA plans, 28.2% through VHA-direct care, and 41.3% through VHA-paid community care (Figure 1). In contrast, among VHA enrollees in other MA plans, 56.2% of surgical episodes were paid by MA plans, 17.2% through VHA-direct care, and 26.6% through VHA-paid community care (Figure 1). Compared with those in other MA plans, VHA enrollees in high-veteran MA plans were significantly less likely to have their surgical episodes paid by MA plans (adjusted difference, −25.7 percentage points; 95% CI, −26.7 to −24.6 percentage points) and more likely to receive their surgery covered through VHA-direct care (adjusted difference, 11.0 percentage points; 95% CI, 10.0-12.0 percentage points) or VHA-paid community care (adjusted difference, 14.7 percentage points; 95% CI, 13.6-15.8 percentage points) (Figure 2).

### Payment Sources by Surgical Complexity

As the complexity of surgical episodes increased, VHA enrollees were more likely to receive surgical care through the community (either funded by MA plans or the VHA-paid community care). Specifically, while 27.0% of surgical episodes involving the least complex procedures (ie, lowest DRG weight quintile) utilized VHA-paid direct care, this proportion decreased to 12.1% for surgeries involving the most complex procedures (ie, highest DRG weight quintile) (Table 2). Relatedly, the gap in the use of VHA-paid direct care between high-veteran MA plans and other MA plans narrowed as the complexity of surgeries increased. VHA enrollees in high-veteran MA plans were more likely to receive surgical care in VHA facilities for the least complex surgeries than those in other MA plans (15.8 percentage points; 95% CI, 12.3-19.3 percentage points); however, this difference narrowed for the most complex surgeries (5.8 percentage points; 95% CI, 4.1-7.4 percentage points) (Figure 2; eTable 3 in Supplement 1). Meanwhile, the gap in the use of VHA-paid community care between high-veteran MA plans and other MA plans widened with surgical complexity (Figure 2; eTable 3 in Supplement 1).

**Table 2.** Adjusted Inpatient Surgery Payment Sources for VHA Enrollees by Surgical Complexity and Source of Admission, 2021^a^

Classification	Estimate, % (95% CI)^b^		
	MA-paid community care	VHA-paid community care	VHA-paid direct care
By surgical complexity			
Q1 (least complex)	48.6 (47.4-49.9)	24.4 (23.3-25.5)	27.0 (25.8-28.1)
Q2	51.6 (50.5-52.7)	27.9 (26.9-28.9)	20.4 (19.5-21.4)
Q3	53.9 (53.0-54.9)	29.9 (29.0-30.8)	16.1 (15.4-16.9)
Q4	51.6 (51.0-52.3)	25.9 (25.3-26.5)	22.4 (21.9-23.0)
Q5 (most complex)	53.2 (52.5-54.0)	34.7 (34.0-35.4)	12.1 (11.6-12.6)
By source of admission			
Elective	49.2 (48.7-49.8)	27.5 (27.0-28.0)	23.3 (22.8-23.7)
Nonelective	55.7 (55.1-56.3)	31.4 (30.8-32.0)	12.9 (12.5-13.3)

Abbreviations: MA, Medicare Advantage; Q, quintile; VHA, Veterans Health Administration.

^a^ Separate multinomial logistic regression models were estimated for surgical complexity and source of admission. The dependent variable is the payment source of the surgical episode, and the key explanatory variable is surgical complexity/source of admission.

^b^ Adjusted share represents the proportion of surgical episodes that are covered by MA plans, VHA-paid community care, and VHA-paid direct care after accounting for VHA enrollees’ age, gender, race and ethnicity, Medicare and Medicaid dual eligibility, rurality, priority group designations, and state fixed effects.

### Payment Sources by Source of Admission

Compared with elective episodes, nonelective surgical episodes were overall more likely to take place in the community (87.1% vs 76.7%) and less likely to take place in VHA direct care facilities (12.9% vs 23.3%) (Table 2). Additionally, the differences in payment sources between high-veteran MA plans and other MA plans were less pronounced for nonelective surgeries compared with elective surgeries. For example, for elective surgical episodes, VHA enrollees in high-veteran MA plans were more likely to be covered through VHA-paid direct care compared with those in other MA plans (13.2 percentage points; 95% CI, 11.9-14.6 percentage points). In contrast, for nonelective surgical episodes, such difference decreased to 7.1 percentage points (95% CI, 5.7-8.5 percentage points) (Figure 2; eTable 3 in Supplement 1).

### Sensitivity Analyses

In sensitivity analyses, using 2-part logistic regression model, we found VHA enrollees in high-veteran MA plans were significantly more likely to use VHA-paid surgical care compared with those in other MA plans (eTable 4 in Supplement 1). Among surgical episodes paid by the VHA, however, we did not find a significant difference between high-veteran MA plans vs other MA plans in using VHA-paid direct care vs VHA-paid community care (eTable 4 in Supplement 1). Additionally, when limiting analyses to MA plans with high data completeness, we found the results remain largely unchanged (eTable 5 in Supplement 1).

## Discussion

In 2021, veterans enrolled in high-veteran MA plans were significantly less likely to have their surgical care paid by MA plans compared with veterans in other MA plans; instead, these VHA enrollees were more likely to rely on the VHA for payment of surgical care, either through VHA-paid community care or VHA-paid direct care. Overall, these findings raise questions about the potential for duplicative spending by the federal government. Specifically, when veterans enrolled in MA plans also seek inpatient surgical care through the VHA, the federal government ends up effectively paying twice, both for the care financed by the VHA and the full capitation rates to the MA plans regardless of the actual volume of health care services provided by the plan.

Our findings were consistent across surgical procedures of varying surgical complexity and source of admission. While VHA enrollees were more likely to receive surgical care in the community for surgical episodes of higher complexity, the difference in the use of VHA-paid community care was wider among high-veteran MA plans than other MA plans for high-risk surgeries compared with lower risk surgeries. Considering known trends of veterans increasingly receiving high risk surgery in the community rather than VHA facilities,^31,32,33,34^ these results suggest that the high costs of complex surgery may continue to be shifted to the VHA if the enrollment growth in high-veteran MA plans continues. Additionally, it may be helpful if the VHA creates referral hubs that centralize resources and expertise for complex surgeries, which could help enhance the VHA’s capacity to manage high-risk procedures, reduce care fragmentation, and lower overall costs.

There are multiple potential explanations for the observed differences in payment sources for inpatient surgical episodes. High-veteran MA plans may preferentially enroll veterans who are more likely to use the VHA as the payer. Prior research shows that high-veteran MA plans tend to enroll veterans from highest priority group who face zero cost sharing when receiving care paid by the VHA, either at VHA facilities or in the community.^4^ However, these veterans from the highest priority group may face stricter prior authorization, referral requirements, or out-of-pocket costs if MA were to cover their surgical procedure. Prior work has found that MA plans use aggressive utilization management strategies and maintain narrow networks to control utilization of health care services,^35,36,37,38^ including surgical care.^39,40,41,42,43^ It is possible that such administrative barriers may encourage veterans and hospitals to use veterans’ VHA benefits for surgical care. Additionally, community hospitals may opt to bill the VHA system over MA plans for surgical care if the former reimburses at a higher rate than the latter. Since the VHA typically reimburses community hospitals at the Medicare fee-for-service rate,^44^ in instances where MA plans pay less for specific hospital services community hospitals may preferentially bill the VHA. The extent to which each of these mechanisms affected our findings, however, is unknown and necessitates further inquiry.

Our findings have several policy implications. First, to improve the financial efficiency of caring for veterans, policymakers could consider legislative efforts that allow the VHA to bill MA plans for services that fall under Medicare’s purview, which can reduce duplicative spending.^1^ However, MA plans and the VHA each manage their own networks, which complicates billing for shared services and raises questions about who would be ultimately responsible for managing and paying for a veteran’s care. Alternatively, CMS and the VHA could consider a system that establishes MA plans as the primary payer for any care that occurs in community hospitals, and the VHA can serve as the secondary payer to help primarily with cost-sharing for inpatient services. Currently, such an arrangement already exists between the Medicare and the Medicaid program for those who are dually eligible for both programs. Finally, CMS could also consider lowering the capitation rates paid to MA plans for veterans who predominantly get their care at the VHA.^4^ This approach would need to be carefully designed, however, to avoid disincentivizing MA plans from enrolling veterans who may prefer Medicare over VHA care.

### Limitations

This study has limitations. First, our analysis focused on payment sources rather than the actual costs incurred by each source. This is because VHA direct care is funded through a budgetary allocation without specific prices for each surgical procedure, and MA operates on a capitation basis, which precludes detailed accounting of CMS payments for individual surgeries. Additionally, the MA encounter data lack information on actual prices paid by MA plans, thus preventing us from quantifying the profitability of MA plans shifting payment sources. Future research could consider examining the financial implications of the payment source shift. Second, unmeasured confounding at veteran-level and plan-level may bias our estimates. However, basic accounting shows that MA plans are paid for a significant amount of surgical care that is financed by the VHA. Third, although our study only used 2021 data and did not evaluate long-term payment trends, the growing enrollment of veterans in MA indicates that these plans are likely receiving higher revenue relative to costs of the veterans they cover. Finally, while the Research Triangle Institute’s race and ethnicity coding is prone to misclassification,^45^ we opted for this classification due to higher incidence of missing data in the self-reported race/ethnicity data from the VHA Corporate Data Warehouse.

## Conclusions

Results of this cross-sectional study suggest there is substantial variation in the payment sources for surgical care among veterans dually enrolled in VHA care and MA plans. Notably, VHA enrollees in high-veteran MA plans are significantly less likely to have their surgeries paid by MA compared with those in other MA plans, raising questions of cost shifting. These findings underscore the urgent need for targeted policy interventions to ensure more efficient allocation of federal resources when caring for veterans.

## Notes

Supplement 1. **eTable 1.** Veteran Priority Group Designation **eTable 2.** List of Diagnosis Related Group Codes for Identifying Surgical Procedures **eFigure 1.** Proportion of VA-Enrollees in HVMA Plans vs Proportion of VA-Enrollees Among All Medicare Beneficiaries in Plan Service Area, 2021 **eFigure 2.** Unadjusted Comparison of Inpatient Surgery Payment Sources for VA Enrollees in HVMA Plans and Other MA Plans, 2021 **eTable 3.** Adjusted Comparison of Inpatient Surgery Payment Sources for VA Enrollees in HVMA Plans and Other MA Plans, 2021 **eTable 4.** Adjusted Comparison of Inpatient Surgery Payment Sources for VA Enrollees in HVMA Plans and Other MA Plans Using Logistic Regressions, 2021 **eTable 5.** Adjusted Comparison of Inpatient Surgery Payment Sources for VA Enrollees in HVMA Plans and Other MA Plans, Plans with High Data Completeness Only, 2021

Supplement 2.
Data Sharing Statement

## References

[1] Trivedi AN, Grebla RC, Jiang L, Yoon J, Mor V, Kizer KW. Duplicate federal payments for dual enrollees in Medicare Advantage plans and the Veterans Affairs health care system. JAMA. 2012;308(1):67-72. doi:10.1001/jama.2012.7115 22735360 PMC4772733

[2] Meyers DJ, Schwartz AL, Jiang L, Yoon J, Trivedi AN. Spending by the Veterans Health Administration for Medicare Advantage Dual Enrollees, 2011-2020. JAMA. 2024;332(16):1392-1394. doi:10.1001/jama.2024.18073 39356518 PMC11447630

[3] Wagner TH, Schmidt A, Belli F, . Health insurance enrollment among US veterans, 2010-2021. JAMA Netw Open. 2024;7(8):e2430205-e2430205. doi:10.1001/jamanetworkopen.2024.30205 39186266 PMC11514437

[4] Ma Y, Phelan J, Jeong KY, . Medicare Advantage plans with high numbers of veterans: enrollment, utilization, and potential wasteful spending. Health Aff (Millwood). 2024;43(11):1508-1517. doi:10.1377/hlthaff.2024.00302 39496081

[5] Westat. 2022 Survey of Veteran Enrollees’ Health and Use of Health Care Findings Report. 2022. Accessed May 14, 2025. https://www.va.gov/VHASTRATEGY/SOE2022/VASOE-FindingsReport-Final.pdf

[6] Government Accountability Office. Medicare Advantage: action needed to ensure appropriate payments for veterans and nonveterans. 2016. Accessed May 14, 2025. https://www.gao.gov/assets/gao-16-137.pdf

[7] Dayoub EJ, Medvedeva EL, Khatana SAM, Nathan AS, Epstein AJ, Groeneveld PW. Federal payments for coronary revascularization procedures among dual enrollees in Medicare Advantage and the Veterans Affairs Health Care System. JAMA Netw Open. 2020;3(4):e201451. doi:10.1001/jamanetworkopen.2020.1451 32250432 PMC7136831

[8] Grimm C. Medicare could have saved up to $128 million over the course of five years if CMS had implemented controls to address duplicate payments for services provided to individuals with Medicare and Veterans Health Administration benefits. 2023. Accessed May 14, 2025. https://oig.hhs.gov/documents/audit/9651/A-09-22-03004-Complete%20Report.pdf

[9] Passman LJ, Garcia RE, Campbell L, Winter E. Elderly veterans receiving care at a Veterans Affairs Medical Center while enrolled in Medicare-financed HMOs: is the taxpayer paying twice? J Gen Intern Med. 1997;12(4):247-249. 9127230 10.1046/j.1525-1497.1997.012004247.x PMC1497097

[10] Kizer KW, Ibrahim S. Medicare to Veterans Affairs cost shifting-a challenging conundrum. JAMA Health Forum. 2024;5(12):e244319. doi:10.1001/jamahealthforum.2024.4319 39729306

[11] Social Security Administration. Compilation of the Social Security Laws. Accessed May 14, 2025. https://www.ssa.gov/OP_Home/ssact/title18/1862.htm

[12] US Senate. 117th Congress of the US, second session. The VA Mission Act of 2018, S. 2373.

[13] Vashi AA, Urech T, Wu S, Tran LD. Community Emergency Care Use by Veterans in an Era of Expanding Choice. JAMA Netw Open. 2024;7(3):e241626. doi:10.1001/jamanetworkopen.2024.1626 38457180 PMC10924239

[14] Burke LG, Ma Y, Phelan J, . Cost Shifting for Emergency Care of Veterans With Medicare After MISSION Act Implementation. JAMA Health Forum. 2024;5(12):e244312. doi:10.1001/jamahealthforum.2024.4312 39729304 PMC11681371

[15] Humana. Medicare Advantage plans and veterans’ benefits. Accessed 10/17/24, 2024. https://www.humana.com/medicare/veterans

[16] Medicare Plans AARP. Medicare Advantage Plans with veterans in mind. UnitedHealthcare. https://www.aarpmedicareplans.com/shop/medicare-advantage-veteran-plan.html

[17] Dorneo A, Ma Y, Garrido MM, . Characteristics and Benefit Design of Veteran Medicare Advantage Affinity Plans. JAMA Health Forum. 2025;6(3):e250159. doi:10.1001/jamahealthforum.2025.0159 40152874 PMC11953753

[18] Beckman AL, Ryan AM, Figueroa JF. The rise and risks of Medicare Advantage “Affinity Plans”. JAMA. 2024;331(15):1271-1272. doi:10.1001/jama.2024.1703 38506818

[19] Maremont M, Weaver C, McGinty T. Insurers Collected Billions From Medicare for Veterans Who Cost Them Almost Nothing. The Wall Street Journal. Dec 2, 2024. Accessed May 14, 2025. https://www.wsj.com/health/healthcare/veterans-medicare-insurers-collect-billions-bfd47d27

[20] Muñoz E, Muñoz W III, Wise L. National and surgical health care expenditures, 2005-2025. Ann Surg. 2010;251(2):195-200. doi:10.1097/SLA.0b013e3181cbcc9a 20054269

[21] Kaye DR, Luckenbaugh AN, Oerline M, . Understanding the Costs Associated With Surgical Care Delivery in the Medicare Population. Ann Surg. 2020;271(1):23-28. doi:10.1097/SLA.0000000000003165 30601252 PMC6586534

[22] George EL, Massarweh NN, Youk A, . Comparing Veterans Affairs and Private Sector Perioperative Outcomes After Noncardiac Surgery. JAMA Surg. 2022;157(3):231-239. doi:10.1001/jamasurg.2021.6488 34964818 PMC8717209

[23] Barnett PG, Hong JS, Carey E, Grunwald GK, Joynt Maddox K, Maddox TM. Comparison of Accessibility, Cost, and Quality of Elective Coronary Revascularization Between Veterans Affairs and Community Care Hospitals. JAMA Cardiol. 2018;3(2):133-141. doi:10.1001/jamacardio.2017.4843 29299607 PMC5838592

[24] Wagner TH, Lo J, Beilstein-Wedel E, Vanneman ME, Shwartz M, Rosen AK. Estimating the Cost of Surgical Care Purchased in the Community by the Veterans Health Administration. MDM Policy Pract. 2021;6(2):23814683211057902. doi:10.1177/23814683211057902 34820527 PMC8606928

[25] US Department of Veterans Affairs Information Resource Center. VIReC Research User Guide: PSSG Geocoded Enrollee Files, 2015 Edition. US Department of Veterans Affairs, Health Services Research & Development Service, Information Resource Center; May 2016.

[26] Centers for Medicare and Medicaid Services. Master Beneficiary Summary File (MBSF). ResDAC. 2024. Accessed May 14, 2025. https://resdac.org/cms-data/files/mbsf-base

[27] Department of Veterans Affairs. 2023 VA health care copay rates. VA. 2024. Accessed May 14, 2025. https://www.va.gov/health-care/copay-rates/

[28] Centers for Medicare and Medicaid Services. FY 2021 Final Rule Tables and Correction Notice Tables - Table 5 (FY 2021 Final Rule and Correction Notice MS-DRGs, Relative Weighting Factors and Geometric and Arithmetic Mean Length of Stay). Accessed May 14, 2025. https://www.cms.gov/medicare/payment/prospective-payment-systems/acute-inpatient-pps/fy-2021-ipps-final-rule-home-page#Tables

[29] Joiner KA, Lin J, Pantano J. Upcoding in Medicare: where does it matter most? Health Econ Rev. 2024;14(1):1. doi:10.1186/s13561-023-00465-4 38165452 PMC10759668

[30] Jung J, Carlin C, Feldman R, Tran L. Implementation of resource use measures in Medicare Advantage. Health Serv Res. 2022;57(4):957-962. doi:10.1111/1475-6773.13970 35411550 PMC10501335

[31] Mull HJ, Kabdiyeva A, Ndugga N, Gordon SH, Garrido MM, Pizer SD. What is the role of selection bias in quality comparisons between the Veterans Health Administration and community care? Example of elective hernia surgery. Health Serv Res. 2023;58(3):654-662. doi:10.1111/1475-6773.14113 36477645 PMC10154155

[32] Rosen AK, O’Brien W, Chen Q, Shwartz M, Itani KFM, Gunnar W. Trends in the Purchase of Surgical Care in the Community by the Veterans Health Administration. Med Care. 2017;55(7)(Suppl 7 Suppl 1):S45-S52. doi:10.1097/MLR.0000000000000707 28319582

[33] Graham LA, Schoemaker L, Rose L, Morris AM, Aouad M, Wagner TH. Expansion of the Veterans Health Administration Network and Surgical Outcomes. JAMA Surg. 2022;157(12):1115-1123. doi:10.1001/jamasurg.2022.4978 36223115 PMC9558067

[34] Rosen AK, Vanneman ME, O’Brien WJ, . Comparing cataract surgery complication rates in veterans receiving VA and community care. Health Serv Res. 2020;55(5):690-700. doi:10.1111/1475-6773.13320 32715468 PMC7518823

[35] Jacobson G, Rae M, Neuman T, Orgera K, Boccuti C. Medicare Advantage: How Robust Are Plans’ Physician Networks? 2017. Accessed May 14, 2025. https://www.kff.org/report-section/medicare-advantage-how-robust-are-plans-physician-networks-report/

[36] Loomer L, Kosar CM, Meyers DJ, Thomas KS. Comparing receipt of prescribed post-acute home health care between Medicare Advantage and Traditional Medicare beneficiaries: an observational study. J Gen Intern Med. 2021;36(8):2323-2331. doi:10.1007/s11606-020-06282-3 33051838 PMC8342740

[37] Gupta R, Fein J, Newhouse JP, Schwartz AL. Comparison of prior authorization across insurers: cross sectional evidence from Medicare Advantage. BMJ. 2024;384:e077797. doi:10.1136/bmj-2023-077797 38453187 PMC10919211

[38] Anderson KE, Alexander GC, Ma C, Dy SM, Sen AP. Medicare Advantage coverage restrictions for the costliest physician-administered drugs. Am J Manag Care. 2022;28(7):e255-e262. doi:10.37765/ajmc.2022.89184 35852888 PMC11370996

[39] Anderson KE, Wu RJ, Darden M, Jain A. Medicare Advantage is associated with lower utilization of total joint arthroplasty. J Bone Joint Surg Am. 2024;106(3):198-205. doi:10.2106/JBJS.23.00507 37973049 PMC11376029

[40] Schwartz AL, Brennan TA, Verbrugge DJ, Newhouse JP. Measuring the scope of prior authorization policies: applying private insurer rules to Medicare Part B. JAMA Health Forum. 2021;2(5):e210859. doi:10.1001/jamahealthforum.2021.0859 35977311 PMC8796979

[41] Landon BE, Zaslavsky AM, Saunders RC, Pawlson LG, Newhouse JP, Ayanian JZ. Analysis Of Medicare Advantage HMOs compared with traditional Medicare shows lower use of many services during 2003-09. Health Aff (Millwood). 2012;31(12):2609-2617. doi:10.1377/hlthaff.2012.0179 23213144 PMC3587962

[42] Curto V, Einav L, Finkelstein A, Levin J, Bhattacharya J. Health care spending and utilization in public and private Medicare. Am Econ J Appl Econ. 2019;11(2):302-332. doi:10.1257/app.20170295 31131073 PMC6532061

[43] Grimm C. Some Medicare Advantage Organization Denials of Prior Authorization Requests Raise Concerns About Beneficiary Access to Medically Necessary Care. 2022. Accessed May 14, 2025. https://oig.hhs.gov/documents/evaluation/3150/OEI-09-18-00260-Complete%20Report.pdf

[44] U.S. Department of Veterans Affairs. VA Fee Schedule. Accessed May 14, 2025. https://www.va.gov/COMMUNITYCARE/revenue-ops/Fee-Schedule.asp

[45] Jarrín OF, Nyandege AN, Grafova IB, Dong X, Lin H. Validity of race and ethnicity codes in Medicare administrative data compared with gold-standard self-reported race collected during routine home health care visits. Med Care. 2020;58(1):e1-e8. doi:10.1097/MLR.0000000000001216 31688554 PMC6904433

