# Supplementary material for: Payment Source Shift for Surgical Care Among Veterans Enrolled in Medicare Advantage Plans
Source: JAMA Health Forum. 2025 Jun 9;6(6):e250827. doi: 10.1001/jamahealthforum.2025.0827 (PMC12150190; doi:10.1001/jamahealthforum.2025.0827)
Supplement: Supplement 1. — eTable 1. Veteran Priority Group Designation eTable 2. List of Diagnosis Related Group Codes for Identifying Surgical Procedures eFigure 1. Proportion of VA-Enrollees in HVMA Plans vs Proportion of VA-Enrollees Among All Medicare Beneficiaries in Plan Service Area, 2021 eFigure 2. Unadjusted Comparison of Inpatient Surgery Payment Sources for VA Enrollees in HVMA Plans and Other MA Plans, 2021 eTable 3. Adjusted Comparison of Inpatient Surgery Payment Sources for VA Enrollees in HVMA Plans and Other MA Plans, 2021 eTable 4. Adjusted Comparison of Inpatient Surgery Payment Sources for VA Enrollees in HVMA Plans and Other MA Plans Using Logistic Regressions, 2021 eTable 5. Adjusted Comparison of Inpatient Surgery Payment Sources for VA Enrollees in HVMA Plans and Other MA Plans, Plans with High Data Completeness Only, 2021 [file jamahealthforum-e250827-s001.pdf]

Supplemental Online Content

Mehtsun WT, Ma Y, Latsko E, et al. Payment source shift for surgical care among veterans enrolled in Medicare Advantage plans. *JAMA Health Forum.* Published online June 9, 2025. doi:10.1001/jamahealthforum.2025.0827

eTable 1. Veteran Priority Group Designation

eTable 2. List of Diagnosis Related Group Codes for Identifying Surgical Procedures

eFigure 1. Proportion of VA-Enrollees in HVMA Plans vs. Proportion of VA-Enrollees Among All Medicare Beneficiaries in Plan Service Area, 2021

eFigure 2. Unadjusted Comparison of Inpatient Surgery Payment Sources for VA Enrollees in HVMA Plans and Other MA Plans, 2021

eTable 3. Adjusted Comparison of Inpatient Surgery Payment Sources for VA Enrollees in HVMA Plans and Other MA Plans, 2021

eTable 4. Adjusted Comparison of Inpatient Surgery Payment Sources for VA Enrollees in HVMA Plans and Other MA Plans Using Logistic Regressions, 2021

eTable 5. Adjusted Comparison of Inpatient Surgery Payment Sources for VA Enrollees in HVMA Plans and Other MA Plans, Plans with High Data Completeness Only, 2021

This supplemental material has been provided by the authors to give readers additional information about their work.

© 2025 Mehtsun WT et al. *JAMA Health Forum*.

eTable 1. Veteran Priority Group Designation

Priority Group	Descriptions
1	Veterans with a VA-rated service-connected disability that is rated by the VA as 50% or more disabling, or<br>Veterans determined by the VA to be unemployable due to a service-connected condition, or<br>Veterans who have received the Medal of Honor
2	Veterans with VA-rated service-connected disabilities that are 30% or 40% disabling
3	Veterans who are a former prisoner of war (POW), or<br>Veterans received the Purple Heart medal, or<br>Veterans who were discharged for a disability that was caused by—or got worse because of— active-duty service, or<br>Veterans with a VA-rated service-connected disability rated as 10% or 20% disabling, or<br>Veterans who were awarded special eligibility classification under Title 38, U.S.C § 1151, “benefits for individuals disabled by treatment or vocational rehabilitation”
4	Veterans who are receiving VA aid and attendance or housebound benefits, or<br>Veterans who have received a VA determination of being catastrophically disabled
5	Veterans without a service-connected disability, or Veterans with a non-compensable service-connected disability rated as 0% disabling, and whose annual income level are below the VA pension benefits national income threshold, or<br>Veterans receiving VA pension benefits, or<br>Veterans eligible for Medicaid programs
6	Veterans with a compensable service-connected disability rated as 0% disabling, or<br>Veterans exposed to ionizing radiation during atmospheric testing or during the occupation of Hiroshima and Nagasaki, or<br>Veterans who participated in Project 112/SHAD, or<br>Veterans who served in World War II between December 7, 1941, and December 31, 1946, or<br>Veterans who served in the Republic of Vietnam between January 9, 1962, and May 7, 1975, or<br>Veterans who served in the Persian Gulf War between August 2, 1990, and November 11, 1998, or<br>Veterans who served on active duty at Camp Lejeune for at least 30 days between August 1, 1953, and December 31, 1987, or<br>Veterans who meet all three of the following requirements:<br>1) You’re currently or newly enrolled in VA health care, and<br>2) You served in a theater of combat operations after November 11, 1998, and<br>3) You were discharged less than 10 years ago
7A	Veterans with income below the geographically adjusted income limits who agree to pay copays, and with a non-compensable service-connected condition rated as 0% disabling who were enrolled a specified date and who have remained enrolled since that date
7C	Veterans with income below the geographically adjusted income limits who agree to pay copays, and with a non-service-connected condition who were enrolled a specified date and who have remained enrolled since that date
8A	Veterans with income above the VA national income threshold and the geographic income threshold who agree to pay co-pays, and<br>with a non-compensable service-connected condition rated 0% disabling enrolled as of January 16, 2003, and who have remained enrolled since that date
8B	Veterans with income above the VA national income threshold and the geographic income threshold who agree to pay co-pays, and<br>with non-compensable service-connected condition rated 0% disabling, who are enrolled in the VA health care program on or after June 15, 2009 and who have income that exceeds the current VA or geographical limits by 10% or less
8C	Veterans with income above the VA national income threshold and the geographic income threshold who agree to pay co-pays, and<br>with a non-service-connected condition enrolled in the VA health care program as of January 16, 2003, and who have remained enrolled since that date
8D	Veterans with income above the VA national income threshold and the geographic income threshold who agree to pay co-pays, and<br>with a non-service-connected condition enrolled in the VA health care program on or after June 15, 2009 and have income higher than the current VA or geographical limits by 10% or less

Source: U.S. Department of Veterans Affairs. VA priority groups. VA Benefits and Health Care. U.S. Department of Veterans Affairs. Published December 5, 2023. Accessed December 19, 2023.

© 2025 Mehtsun WT et al. *JAMA Health Forum*.

eTable 2. List of Diagnosis Related Group Codes for Identifying Surgical Procedures

DRG	Title	Base DRG Weight	Base DRG Quintile
**1**	HEART TRANSPLANT OR IMPLANT OF HEART ASSIST SYSTEM WITH MCC	15.931	5
**2**	HEART TRANSPLANT OR IMPLANT OF HEART ASSIST SYSTEM WITHOUT MCC	15.931	5
**3**	ECMO OR TRACHEOSTOMY WITH MV >96 HOURS OR PRINCIPAL DIAGNOSIS EXCEPT FACE, MOUTH AND NECK WITH MAJOR O.R. PROCEDURES	18.9911	5
**4**	TRACHEOSTOMY WITH MV >96 HOURS OR PRINCIPAL DIAGNOSIS EXCEPT FACE, MOUTH AND NECK WITHOUT MAJOR O.R. PROCEDURES	11.862	5
**5**	LIVER TRANSPLANT WITH MCC OR INTESTINAL TRANSPLANT	4.6917	5
**6**	LIVER TRANSPLANT WITHOUT MCC	4.6917	5
**7**	LUNG TRANSPLANT	11.5743	5
**8**	SIMULTANEOUS PANCREAS AND KIDNEY TRANSPLANT	5.4268	5
**10**	PANCREAS TRANSPLANT	3.6177	5
**11**	TRACHEOSTOMY FOR FACE, MOUTH AND NECK DIAGNOSES OR LARYNGECTOMY WITH MCC	2.7205	5
**12**	TRACHEOSTOMY FOR FACE, MOUTH AND NECK DIAGNOSES OR LARYNGECTOMY WITH CC	2.7205	5
**13**	TRACHEOSTOMY FOR FACE, MOUTH AND NECK DIAGNOSES OR LARYNGECTOMY WITHOUT CC/MCC	2.7205	5
**19**	SIMULTANEOUS PANCREAS AND KIDNEY TRANSPLANT WITH HEMODIALYSIS	6.6619	5
**20**	INTRACRANIAL VASCULAR PROCEDURES WITH PRINCIPAL DIAGNOSIS HEMORRHAGE WITH MCC	4.8596	5
**21**	INTRACRANIAL VASCULAR PROCEDURES WITH PRINCIPAL DIAGNOSIS HEMORRHAGE WITH CC	4.8596	5
**22**	INTRACRANIAL VASCULAR PROCEDURES WITH PRINCIPAL DIAGNOSIS HEMORRHAGE WITHOUT CC/MCC	4.8596	5
**23**	CRANIOTOMY WITH MAJOR DEVICE IMPLANT OR ACUTE COMPLEX CNS PRINCIPAL DIAGNOSIS WITH MCC OR CHEMOTHERAPY IMPLANT OR EPILEPSY WITH NEUROSTIMULATOR	3.9325	5
**24**	CRANIOTOMY WITH MAJOR DEVICE IMPLANT OR ACUTE COMPLEX CNS PRINCIPAL DIAGNOSIS WITHOUT MCC	3.9325	5
**25**	CRANIOTOMY AND ENDOVASCULAR INTRACRANIAL PROCEDURES WITH MCC	2.5118	5
**26**	CRANIOTOMY AND ENDOVASCULAR INTRACRANIAL PROCEDURES WITH CC	2.5118	5
**27**	CRANIOTOMY AND ENDOVASCULAR INTRACRANIAL PROCEDURES WITHOUT CC/MCC	2.5118	5
**28**	SPINAL PROCEDURES WITH MCC	2.3571	4
**29**	SPINAL PROCEDURES WITH CC OR SPINAL NEUROSTIMULATORS	2.3571	4
**30**	SPINAL PROCEDURES WITHOUT CC/MCC	2.3571	4
**31**	VENTRICULAR SHUNT PROCEDURES WITH MCC	1.7207	4
**32**	VENTRICULAR SHUNT PROCEDURES WITH CC	1.7207	4
**33**	VENTRICULAR SHUNT PROCEDURES WITHOUT CC/MCC	1.7207	4
**34**	CAROTID ARTERY STENT PROCEDURES WITH MCC	1.8512	4

© 2025 Mehtsun WT et al. *JAMA Health Forum*.

**35**	CAROTID ARTERY STENT PROCEDURES WITH CC	1.8512	4
**36**	CAROTID ARTERY STENT PROCEDURES WITHOUT CC/MCC	1.8512	4
**37**	EXTRACRANIAL PROCEDURES WITH MCC	1.139	1
**38**	EXTRACRANIAL PROCEDURES WITH CC	1.139	1
**39**	EXTRACRANIAL PROCEDURES WITHOUT CC/MCC	1.139	1
**40**	PERIPHERAL, CRANIAL NERVE AND OTHER NERVOUS SYSTEM PROCEDURES WITH MCC	1.8849	4
**41**	PERIPHERAL, CRANIAL NERVE AND OTHER NERVOUS SYSTEM PROCEDURES WITH CC OR PERIPHERAL NEUROSTIMULATOR	1.8849	4
**42**	PERIPHERAL, CRANIAL NERVE AND OTHER NERVOUS SYSTEM PROCEDURES WITHOUT CC/MCC	1.8849	4
**113**	ORBITAL PROCEDURES WITH CC/MCC	1.4455	3
**114**	ORBITAL PROCEDURES WITHOUT CC/MCC	1.4455	3
**115**	EXTRAOCULAR PROCEDURES EXCEPT ORBIT	1.4533	3
**116**	INTRAOCULAR PROCEDURES WITH CC/MCC	1.0389	1
**117**	INTRAOCULAR PROCEDURES WITHOUT CC/MCC	1.0389	1
**135**	SINUS AND MASTOID PROCEDURES WITH CC/MCC	1.2555	2
**136**	SINUS AND MASTOID PROCEDURES WITHOUT CC/MCC	1.2555	2
**137**	MOUTH PROCEDURES WITH CC/MCC	0.8431	1
**138**	MOUTH PROCEDURES WITHOUT CC/MCC	0.8431	1
**139**	SALIVARY GLAND PROCEDURES	1.2268	2
**140**	MAJOR HEAD AND NECK PROCEDURES WITH MCC	1.6088	3
**141**	MAJOR HEAD AND NECK PROCEDURES WITH CC	1.6088	3
**142**	MAJOR HEAD AND NECK PROCEDURES WITHOUT CC/MCC	1.6088	3
**143**	OTHER EAR, NOSE, MOUTH AND THROAT O.R. PROCEDURES WITH MCC	1.2135	2
**144**	OTHER EAR, NOSE, MOUTH AND THROAT O.R. PROCEDURES WITH CC	1.2135	2
**145**	OTHER EAR, NOSE, MOUTH AND THROAT O.R. PROCEDURES WITHOUT CC/MCC	1.2135	2
**163**	MAJOR CHEST PROCEDURES WITH MCC	1.9136	4
**164**	MAJOR CHEST PROCEDURES WITH CC	1.9136	4
**165**	MAJOR CHEST PROCEDURES WITHOUT CC/MCC	1.9136	4
**166**	OTHER RESPIRATORY SYSTEM O.R. PROCEDURES WITH MCC	1.3691	2
**167**	OTHER RESPIRATORY SYSTEM O.R. PROCEDURES WITH CC	1.3691	2
**168**	OTHER RESPIRATORY SYSTEM O.R. PROCEDURES WITHOUT CC/MCC	1.3691	2
**215**	OTHER HEART ASSIST SYSTEM IMPLANT	11.1579	5
**216**	CARDIAC VALVE AND OTHER MAJOR CARDIOTHORACIC PROCEDURES WITH CARDIAC CATHETERIZATION WITH MCC	5.1432	5
**217**	CARDIAC VALVE AND OTHER MAJOR CARDIOTHORACIC PROCEDURES WITH CARDIAC CATHETERIZATION WITH CC	5.1432	5
**218**	CARDIAC VALVE AND OTHER MAJOR CARDIOTHORACIC PROCEDURES WITH CARDIAC CATHETERIZATION WITHOUT CC/MCC	5.1432	5
**219**	CARDIAC VALVE AND OTHER MAJOR CARDIOTHORACIC PROCEDURES WITHOUT CARDIAC CATHETERIZATION WITH MCC	4.5523	5
**220**	CARDIAC VALVE AND OTHER MAJOR CARDIOTHORACIC PROCEDURES WITHOUT CARDIAC CATHETERIZATION WITH CC	4.5523	5
**221**	CARDIAC VALVE AND OTHER MAJOR CARDIOTHORACIC PROCEDURES WITHOUT CARDIAC CATHETERIZATION WITHOUT CC/MCC	4.5523	5

© 2025 Mehtsun WT et al. *JAMA Health Forum*.

**229**	OTHER CARDIOTHORACIC PROCEDURES WITHOUT MCC	3.988	5
**235**	CORONARY BYPASS WITHOUT CARDIAC CATHETERIZATION WITH MCC	4.0808	5
**236**	CORONARY BYPASS WITHOUT CARDIAC CATHETERIZATION WITHOUT MCC	4.0808	5
**239**	AMPUTATION FOR CIRCULATORY SYSTEM DISORDERS EXCEPT UPPER LIMB AND TOE WITH MCC	1.5914	3
**240**	AMPUTATION FOR CIRCULATORY SYSTEM DISORDERS EXCEPT UPPER LIMB AND TOE WITH CC	1.5914	3
**241**	AMPUTATION FOR CIRCULATORY SYSTEM DISORDERS EXCEPT UPPER LIMB AND TOE WITHOUT CC/MCC	1.5914	3
**252**	OTHER VASCULAR PROCEDURES WITH MCC	1.8095	4
**253**	OTHER VASCULAR PROCEDURES WITH CC	1.8095	4
**254**	OTHER VASCULAR PROCEDURES WITHOUT CC/MCC	1.8095	4
**255**	UPPER LIMB AND TOE AMPUTATION FOR CIRCULATORY SYSTEM DISORDERS WITH MCC	1.161	1
**256**	UPPER LIMB AND TOE AMPUTATION FOR CIRCULATORY SYSTEM DISORDERS WITH CC	1.161	1
**257**	UPPER LIMB AND TOE AMPUTATION FOR CIRCULATORY SYSTEM DISORDERS WITHOUT CC/MCC	1.161	1
**263**	VEIN LIGATION AND STRIPPING	2.3053	4
**264**	OTHER CIRCULATORY SYSTEM O.R. PROCEDURES	3.2478	5
**266**	ENDOVASCULAR CARDIAC VALVE REPLACEMENT AND SUPPLEMENT PROCEDURES WITH MCC	5.6009	5
**267**	ENDOVASCULAR CARDIAC VALVE REPLACEMENT AND SUPPLEMENT PROCEDURES WITHOUT MCC	5.6009	5
**268**	AORTIC AND HEART ASSIST PROCEDURES EXCEPT PULSATION BALLOON WITH MCC	4.3146	5
**269**	AORTIC AND HEART ASSIST PROCEDURES EXCEPT PULSATION BALLOON WITHOUT MCC	4.3146	5
**270**	OTHER MAJOR CARDIOVASCULAR PROCEDURES WITH MCC	2.6888	5
**271**	OTHER MAJOR CARDIOVASCULAR PROCEDURES WITH CC	2.6888	5
**272**	OTHER MAJOR CARDIOVASCULAR PROCEDURES WITHOUT CC/MCC	2.6888	5
**326**	STOMACH, ESOPHAGEAL AND DUODENAL PROCEDURES WITH MCC	1.6655	4
**327**	STOMACH, ESOPHAGEAL AND DUODENAL PROCEDURES WITH CC	1.6655	4
**328**	STOMACH, ESOPHAGEAL AND DUODENAL PROCEDURES WITHOUT CC/MCC	1.6655	4
**329**	MAJOR SMALL AND LARGE BOWEL PROCEDURES WITH MCC	1.7101	4
**330**	MAJOR SMALL AND LARGE BOWEL PROCEDURES WITH CC	1.7101	4
**331**	MAJOR SMALL AND LARGE BOWEL PROCEDURES WITHOUT CC/MCC	1.7101	4
**332**	RECTAL RESECTION WITH MCC	1.6092	3
**333**	RECTAL RESECTION WITH CC	1.6092	3
**334**	RECTAL RESECTION WITHOUT CC/MCC	1.6092	3
**335**	PERITONEAL ADHESIOLYSIS WITH MCC	1.6326	3
**336**	PERITONEAL ADHESIOLYSIS WITH CC	1.6326	3
**337**	PERITONEAL ADHESIOLYSIS WITHOUT CC/MCC	1.6326	3
**338**	APPENDECTOMY WITH COMPLICATED PRINCIPAL DIAGNOSIS WITH MCC	1.2284	2
**339**	APPENDECTOMY WITH COMPLICATED PRINCIPAL DIAGNOSIS WITH CC	1.2284	2
**340**	APPENDECTOMY WITH COMPLICATED PRINCIPAL DIAGNOSIS WITHOUT CC/MCC	1.2284	2

© 2025 Mehtsun WT et al. *JAMA Health Forum*.

**341**	APPENDECTOMY WITHOUT COMPLICATED PRINCIPAL DIAGNOSIS WITH MCC	1.1094	1
**342**	APPENDECTOMY WITHOUT COMPLICATED PRINCIPAL DIAGNOSIS WITH CC	1.1094	1
**343**	APPENDECTOMY WITHOUT COMPLICATED PRINCIPAL DIAGNOSIS WITHOUT CC/MCC	1.1094	1
**344**	MINOR SMALL AND LARGE BOWEL PROCEDURES WITH MCC	1.275	2
**345**	MINOR SMALL AND LARGE BOWEL PROCEDURES WITH CC	1.275	2
**346**	MINOR SMALL AND LARGE BOWEL PROCEDURES WITHOUT CC/MCC	1.275	2
**347**	ANAL AND STOMAL PROCEDURES WITH MCC	0.9793	1
**348**	ANAL AND STOMAL PROCEDURES WITH CC	0.9793	1
**349**	ANAL AND STOMAL PROCEDURES WITHOUT CC/MCC	0.9793	1
**350**	INGUINAL AND FEMORAL HERNIA PROCEDURES WITH MCC	1.103	1
**351**	INGUINAL AND FEMORAL HERNIA PROCEDURES WITH CC	1.103	1
**352**	INGUINAL AND FEMORAL HERNIA PROCEDURES WITHOUT CC/MCC	1.103	1
**353**	HERNIA PROCEDURES EXCEPT INGUINAL AND FEMORAL WITH MCC	1.3592	2
**354**	HERNIA PROCEDURES EXCEPT INGUINAL AND FEMORAL WITH CC	1.3592	2
**355**	HERNIA PROCEDURES EXCEPT INGUINAL AND FEMORAL WITHOUT CC/MCC	1.3592	2
**356**	OTHER DIGESTIVE SYSTEM O.R. PROCEDURES WITH MCC	1.339	2
**357**	OTHER DIGESTIVE SYSTEM O.R. PROCEDURES WITH CC	1.339	2
**358**	OTHER DIGESTIVE SYSTEM O.R. PROCEDURES WITHOUT CC/MCC	1.339	2
**405**	PANCREAS, LIVER AND SHUNT PROCEDURES WITH MCC	2.116	4
**406**	PANCREAS, LIVER AND SHUNT PROCEDURES WITH CC	2.116	4
**407**	PANCREAS, LIVER AND SHUNT PROCEDURES WITHOUT CC/MCC	2.116	4
**408**	BILIARY TRACT PROCEDURES EXCEPT ONLY CHOLECYSTECTOMY WITH OR WITHOUT C.D.E. WITH MCC	1.5657	3
**409**	BILIARY TRACT PROCEDURES EXCEPT ONLY CHOLECYSTECTOMY WITH OR WITHOUT C.D.E. WITH CC	1.5657	3
**410**	BILIARY TRACT PROCEDURES EXCEPT ONLY CHOLECYSTECTOMY WITH OR WITHOUT C.D.E. WITHOUT CC/MCC	1.5657	3
**411**	CHOLECYSTECTOMY WITH C.D.E. WITH MCC	1.7314	4
**412**	CHOLECYSTECTOMY WITH C.D.E. WITH CC	1.7314	4
**413**	CHOLECYSTECTOMY WITH C.D.E. WITHOUT CC/MCC	1.7314	4
**414**	CHOLECYSTECTOMY EXCEPT BY LAPAROSCOPE WITHOUT C.D.E. WITH MCC	1.4222	3
**415**	CHOLECYSTECTOMY EXCEPT BY LAPAROSCOPE WITHOUT C.D.E. WITH CC	1.4222	3
**416**	CHOLECYSTECTOMY EXCEPT BY LAPAROSCOPE WITHOUT C.D.E. WITHOUT CC/MCC	1.4222	3
**417**	LAPAROSCOPIC CHOLECYSTECTOMY WITHOUT C.D.E. WITH MCC	1.3152	2
**418**	LAPAROSCOPIC CHOLECYSTECTOMY WITHOUT C.D.E. WITH CC	1.3152	2
**419**	LAPAROSCOPIC CHOLECYSTECTOMY WITHOUT C.D.E. WITHOUT CC/MCC	1.3152	2
**420**	HEPATOBILIARY DIAGNOSTIC PROCEDURES WITH MCC	1.4514	3
**421**	HEPATOBILIARY DIAGNOSTIC PROCEDURES WITH CC	1.4514	3
**422**	HEPATOBILIARY DIAGNOSTIC PROCEDURES WITHOUT CC/MCC	1.4514	3
**423**	OTHER HEPATOBILIARY OR PANCREAS O.R. PROCEDURES WITH MCC	1.5433	3
**424**	OTHER HEPATOBILIARY OR PANCREAS O.R. PROCEDURES WITH CC	1.5433	3

© 2025 Mehtsun WT et al. *JAMA Health Forum*.

**425**	OTHER HEPATOBILIARY OR PANCREAS O.R. PROCEDURES WITHOUT CC/MCC	1.5433	3
**453**	COMBINED ANTERIOR AND POSTERIOR SPINAL FUSION WITH MCC	4.791	5
**454**	COMBINED ANTERIOR AND POSTERIOR SPINAL FUSION WITH CC	4.791	5
**455**	COMBINED ANTERIOR AND POSTERIOR SPINAL FUSION WITHOUT CC/MCC	4.791	5
**456**	SPINAL FUSION EXCEPT CERVICAL WITH SPINAL CURVATURE, MALIGNANCY, INFECTION OR EXTENSIVE FUSIONS WITH MCC	5.0174	5
**457**	SPINAL FUSION EXCEPT CERVICAL WITH SPINAL CURVATURE, MALIGNANCY, INFECTION OR EXTENSIVE FUSIONS WITH CC	5.0174	5
**458**	SPINAL FUSION EXCEPT CERVICAL WITH SPINAL CURVATURE, MALIGNANCY, INFECTION OR EXTENSIVE FUSIONS WITHOUT CC/MCC	5.0174	5
**459**	SPINAL FUSION EXCEPT CERVICAL WITH MCC	3.9327	5
**460**	SPINAL FUSION EXCEPT CERVICAL WITHOUT MCC	3.9327	5
**461**	BILATERAL OR MULTIPLE MAJOR JOINT PROCEDURES OF LOWER EXTREMITY WITH MCC	3.1442	5
**462**	BILATERAL OR MULTIPLE MAJOR JOINT PROCEDURES OF LOWER EXTREMITY WITHOUT MCC	3.1442	5
**463**	WOUND DEBRIDEMENT AND SKIN GRAFT EXCEPT HAND FOR MUSCULOSKELETAL AND CONNECTIVE TISSUE DISORDERS WITH MCC	1.8436	4
**464**	WOUND DEBRIDEMENT AND SKIN GRAFT EXCEPT HAND FOR MUSCULOSKELETAL AND CONNECTIVE TISSUE DISORDERS WITH CC	1.8436	4
**465**	WOUND DEBRIDEMENT AND SKIN GRAFT EXCEPT HAND FOR MUSCULOSKELETAL AND CONNECTIVE TISSUE DISORDERS WITHOUT CC/MCC	1.8436	4
**466**	REVISION OF HIP OR KNEE REPLACEMENT WITH MCC	2.804	5
**467**	REVISION OF HIP OR KNEE REPLACEMENT WITH CC	2.804	5
**468**	REVISION OF HIP OR KNEE REPLACEMENT WITHOUT CC/MCC	2.804	5
**469**	MAJOR HIP AND KNEE JOINT REPLACEMENT OR REATTACHMENT OF LOWER EXTREMITY WITH MCC OR TOTAL ANKLE REPLACEMENT	1.8999	4
**470**	MAJOR HIP AND KNEE JOINT REPLACEMENT OR REATTACHMENT OF LOWER EXTREMITY WITHOUT MCC	1.8999	4
**471**	CERVICAL SPINAL FUSION WITH MCC	2.5402	5
**473**	CERVICAL SPINAL FUSION WITHOUT CC/MCC	2.5402	5
**474**	AMPUTATION FOR MUSCULOSKELETAL SYSTEM AND CONNECTIVE TISSUE DISORDERS WITH MCC	1.1598	1
**475**	AMPUTATION FOR MUSCULOSKELETAL SYSTEM AND CONNECTIVE TISSUE DISORDERS WITH CC	1.1598	1
**476**	AMPUTATION FOR MUSCULOSKELETAL SYSTEM AND CONNECTIVE TISSUE DISORDERS WITHOUT CC/MCC	1.1598	1
**477**	BIOPSIES OF MUSCULOSKELETAL SYSTEM AND CONNECTIVE TISSUE WITH MCC	1.8093	4
**478**	BIOPSIES OF MUSCULOSKELETAL SYSTEM AND CONNECTIVE TISSUE WITH CC	1.8093	4
**479**	BIOPSIES OF MUSCULOSKELETAL SYSTEM AND CONNECTIVE TISSUE WITHOUT CC/MCC	1.8093	4
**480**	HIP AND FEMUR PROCEDURES EXCEPT MAJOR JOINT WITH MCC	1.6468	3
**481**	HIP AND FEMUR PROCEDURES EXCEPT MAJOR JOINT WITH CC	1.6468	3
**482**	HIP AND FEMUR PROCEDURES EXCEPT MAJOR JOINT WITHOUT CC/MCC	1.6468	3
**483**	MAJOR JOINT OR LIMB REATTACHMENT PROCEDURES OF UPPER EXTREMITIES	2.3863	5

© 2025 Mehtsun WT et al. *JAMA Health Forum*.

**485**	KNEE PROCEDURES WITH PRINCIPAL DIAGNOSIS OF INFECTION WITH MCC	1.6403	3
**486**	KNEE PROCEDURES WITH PRINCIPAL DIAGNOSIS OF INFECTION WITH CC	1.6403	3
**487**	KNEE PROCEDURES WITH PRINCIPAL DIAGNOSIS OF INFECTION WITHOUT CC/MCC	1.6403	3
**488**	KNEE PROCEDURES WITHOUT PRINCIPAL DIAGNOSIS OF INFECTION WITH CC/MCC	1.2982	2
**489**	KNEE PROCEDURES WITHOUT PRINCIPAL DIAGNOSIS OF INFECTION WITHOUT CC/MCC	1.2982	2
**492**	LOWER EXTREMITY AND HUMERUS PROCEDURES EXCEPT HIP, FOOT AND FEMUR WITH MCC	1.8519	4
**493**	LOWER EXTREMITY AND HUMERUS PROCEDURES EXCEPT HIP, FOOT AND FEMUR WITH CC	1.8519	4
**494**	LOWER EXTREMITY AND HUMERUS PROCEDURES EXCEPT HIP, FOOT AND FEMUR WITHOUT CC/MCC	1.8519	4
**495**	LOCAL EXCISION AND REMOVAL OF INTERNAL FIXATION DEVICES EXCEPT HIP AND FEMUR WITH MCC	1.4587	3
**496**	LOCAL EXCISION AND REMOVAL OF INTERNAL FIXATION DEVICES EXCEPT HIP AND FEMUR WITH CC	1.4587	3
**497**	LOCAL EXCISION AND REMOVAL OF INTERNAL FIXATION DEVICES EXCEPT HIP AND FEMUR WITHOUT CC/MCC	1.4587	3
**498**	LOCAL EXCISION AND REMOVAL OF INTERNAL FIXATION DEVICES OF HIP AND FEMUR WITH CC/MCC	1.1987	2
**499**	LOCAL EXCISION AND REMOVAL OF INTERNAL FIXATION DEVICES OF HIP AND FEMUR WITHOUT CC/MCC	1.1987	2
**500**	SOFT TISSUE PROCEDURES WITH MCC	1.3308	2
**501**	SOFT TISSUE PROCEDURES WITH CC	1.3308	2
**502**	SOFT TISSUE PROCEDURES WITHOUT CC/MCC	1.3308	2
**503**	FOOT PROCEDURES WITH MCC	1.7739	4
**504**	FOOT PROCEDURES WITH CC	1.7739	4
**505**	FOOT PROCEDURES WITHOUT CC/MCC	1.7739	4
**506**	MAJOR THUMB OR JOINT PROCEDURES	1.4757	3
**507**	MAJOR SHOULDER OR ELBOW JOINT PROCEDURES WITH CC/MCC	1.414	2
**508**	MAJOR SHOULDER OR ELBOW JOINT PROCEDURES WITHOUT CC/MCC	1.414	2
**509**	ARTHROSCOPY	1.6738	4
**510**	SHOULDER, ELBOW OR FOREARM PROCEDURES, EXCEPT MAJOR JOINT PROCEDURES WITH MCC	1.5562	3
**511**	SHOULDER, ELBOW OR FOREARM PROCEDURES, EXCEPT MAJOR JOINT PROCEDURES WITH CC	1.5562	3
**512**	SHOULDER, ELBOW OR FOREARM PROCEDURES, EXCEPT MAJOR JOINT PROCEDURES WITHOUT CC/MCC	1.5562	3
**513**	HAND OR WRIST PROCEDURES, EXCEPT MAJOR THUMB OR JOINT PROCEDURES WITH CC/MCC	0.9942	1
**514**	HAND OR WRIST PROCEDURES, EXCEPT MAJOR THUMB OR JOINT PROCEDURES WITHOUT CC/MCC	0.9942	1
**515**	OTHER MUSCULOSKELETAL SYSTEM AND CONNECTIVE TISSUE O.R. PROCEDURES WITH MCC	1.3967	2
**516**	OTHER MUSCULOSKELETAL SYSTEM AND CONNECTIVE TISSUE O.R. PROCEDURES WITH CC	1.3967	2
**517**	OTHER MUSCULOSKELETAL SYSTEM AND CONNECTIVE TISSUE O.R. PROCEDURES WITHOUT CC/MCC	1.3967	2
**518**	BACK AND NECK PROCEDURES EXCEPT SPINAL FUSION WITH MCC OR DISC DEVICE OR NEUROSTIMULATOR	1.4174	3
**519**	BACK AND NECK PROCEDURES EXCEPT SPINAL FUSION WITH CC	1.4174	3

© 2025 Mehtsun WT et al. *JAMA Health Forum*.

**520**	BACK AND NECK PROCEDURES EXCEPT SPINAL FUSION WITHOUT CC/MCC	1.4174	3
**521**	HIP REPLACEMENT WITH PRINCIPAL DIAGNOSIS OF HIP FRACTURE WITH MCC	2.1891	4
**522**	HIP REPLACEMENT WITH PRINCIPAL DIAGNOSIS OF HIP FRACTURE WITHOUT MCC	2.1891	4
**570**	SKIN DEBRIDEMENT WITH MCC	1.1026	1
**571**	SKIN DEBRIDEMENT WITH CC	1.1026	1
**572**	SKIN DEBRIDEMENT WITHOUT CC/MCC	1.1026	1
**573**	SKIN GRAFT FOR SKIN ULCER OR CELLULITIS WITH MCC	1.7615	4
**574**	SKIN GRAFT FOR SKIN ULCER OR CELLULITIS WITH CC	1.7615	4
**575**	SKIN GRAFT FOR SKIN ULCER OR CELLULITIS WITHOUT CC/MCC	1.7615	4
**576**	SKIN GRAFT EXCEPT FOR SKIN ULCER OR CELLULITIS WITH MCC	1.5947	3
**577**	SKIN GRAFT EXCEPT FOR SKIN ULCER OR CELLULITIS WITH CC	1.5947	3
**578**	SKIN GRAFT EXCEPT FOR SKIN ULCER OR CELLULITIS WITHOUT CC/MCC	1.5947	3
**579**	OTHER SKIN, SUBCUTANEOUS TISSUE AND BREAST PROCEDURES WITH MCC	1.2599	2
**580**	OTHER SKIN, SUBCUTANEOUS TISSUE AND BREAST PROCEDURES WITH CC	1.2599	2
**581**	OTHER SKIN, SUBCUTANEOUS TISSUE AND BREAST PROCEDURES WITHOUT CC/MCC	1.2599	2
**582**	MASTECTOMY FOR MALIGNANCY WITH CC/MCC	1.5416	3
**583**	MASTECTOMY FOR MALIGNANCY WITHOUT CC/MCC	1.5416	3
**584**	BREAST BIOPSY, LOCAL EXCISION AND OTHER BREAST PROCEDURES WITH CC/MCC	1.7423	4
**585**	BREAST BIOPSY, LOCAL EXCISION AND OTHER BREAST PROCEDURES WITHOUT CC/MCC	1.7423	4
**614**	ADRENAL AND PITUITARY PROCEDURES WITH CC/MCC	1.5741	3
**615**	ADRENAL AND PITUITARY PROCEDURES WITHOUT CC/MCC	1.5741	3
**616**	AMPUTATION OF LOWER LIMB FOR ENDOCRINE, NUTRITIONAL AND METABOLIC DISORDERS WITH MCC	1.303	2
**617**	AMPUTATION OF LOWER LIMB FOR ENDOCRINE, NUTRITIONAL AND METABOLIC DISORDERS WITH CC	1.303	2
**618**	AMPUTATION OF LOWER LIMB FOR ENDOCRINE, NUTRITIONAL AND METABOLIC DISORDERS WITHOUT CC/MCC	1.303	2
**619**	O.R. PROCEDURES FOR OBESITY WITH MCC	1.5964	3
**620**	O.R. PROCEDURES FOR OBESITY WITH CC	1.5964	3
**621**	O.R. PROCEDURES FOR OBESITY WITHOUT CC/MCC	1.5964	3
**622**	SKIN GRAFTS AND WOUND DEBRIDEMENT FOR ENDOCRINE, NUTRITIONAL AND METABOLIC DISORDERS WITH MCC	1.0943	1
**623**	SKIN GRAFTS AND WOUND DEBRIDEMENT FOR ENDOCRINE, NUTRITIONAL AND METABOLIC DISORDERS WITH CC	1.0943	1
**624**	SKIN GRAFTS AND WOUND DEBRIDEMENT FOR ENDOCRINE, NUTRITIONAL AND METABOLIC DISORDERS WITHOUT CC/MCC	1.0943	1
**625**	THYROID, PARATHYROID AND THYROGLOSSAL PROCEDURES WITH MCC	1.1818	1
**626**	THYROID, PARATHYROID AND THYROGLOSSAL PROCEDURES WITH CC	1.1818	1
**627**	THYROID, PARATHYROID AND THYROGLOSSAL PROCEDURES WITHOUT CC/MCC	1.1818	1
**628**	OTHER ENDOCRINE, NUTRITIONAL AND METABOLIC O.R. PROCEDURES WITH MCC	1.4069	2

© 2025 Mehtsun WT et al. *JAMA Health Forum*.

**629**	OTHER ENDOCRINE, NUTRITIONAL AND METABOLIC O.R. PROCEDURES WITH CC	1.4069	2
**630**	OTHER ENDOCRINE, NUTRITIONAL AND METABOLIC O.R. PROCEDURES WITHOUT CC/MCC	1.4069	2
**650**	KIDNEY TRANSPLANT WITH HEMODIALYSIS WITH MCC	3.1819	5
**651**	KIDNEY TRANSPLANT WITH HEMODIALYSIS WITHOUT MCC	3.1819	5
**652**	KIDNEY TRANSPLANT	3.1819	5
**653**	MAJOR BLADDER PROCEDURES WITH MCC	2.0801	4
**654**	MAJOR BLADDER PROCEDURES WITH CC	2.0801	4
**655**	MAJOR BLADDER PROCEDURES WITHOUT CC/MCC	2.0801	4
**656**	KIDNEY AND URETER PROCEDURES FOR NEOPLASM WITH MCC	1.5791	3
**657**	KIDNEY AND URETER PROCEDURES FOR NEOPLASM WITH CC	1.5791	3
**658**	KIDNEY AND URETER PROCEDURES FOR NEOPLASM WITHOUT CC/MCC	1.5791	3
**659**	KIDNEY AND URETER PROCEDURES FOR NON-NEOPLASM WITH MCC	1.0644	1
**660**	KIDNEY AND URETER PROCEDURES FOR NON-NEOPLASM WITH CC	1.0644	1
**661**	KIDNEY AND URETER PROCEDURES FOR NON-NEOPLASM WITHOUT CC/MCC	1.0644	1
**662**	MINOR BLADDER PROCEDURES WITH MCC	1.1842	1
**663**	MINOR BLADDER PROCEDURES WITH CC	1.1842	1
**664**	MINOR BLADDER PROCEDURES WITHOUT CC/MCC	1.1842	1
**665**	PROSTATECTOMY WITH MCC	0.9949	1
**666**	PROSTATECTOMY WITH CC	0.9949	1
**667**	PROSTATECTOMY WITHOUT CC/MCC	0.9949	1
**668**	TRANSURETHRAL PROCEDURES WITH MCC	0.9765	1
**669**	TRANSURETHRAL PROCEDURES WITH CC	0.9765	1
**670**	TRANSURETHRAL PROCEDURES WITHOUT CC/MCC	0.9765	1
**671**	URETHRAL PROCEDURES WITH CC/MCC	1.1509	1
**672**	URETHRAL PROCEDURES WITHOUT CC/MCC	1.1509	1
**673**	OTHER KIDNEY AND URINARY TRACT PROCEDURES WITH MCC	1.752	4
**674**	OTHER KIDNEY AND URINARY TRACT PROCEDURES WITH CC	1.752	4
**675**	OTHER KIDNEY AND URINARY TRACT PROCEDURES WITHOUT CC/MCC	1.752	4
**707**	MAJOR MALE PELVIC PROCEDURES WITH CC/MCC	1.4914	3
**708**	MAJOR MALE PELVIC PROCEDURES WITHOUT CC/MCC	1.4914	3
**709**	PENIS PROCEDURES WITH CC/MCC	1.5999	3
**710**	PENIS PROCEDURES WITHOUT CC/MCC	1.5999	3
**711**	TESTES PROCEDURES WITH CC/MCC	1.0575	1
**712**	TESTES PROCEDURES WITHOUT CC/MCC	1.0575	1
**713**	TRANSURETHRAL PROSTATECTOMY WITH CC/MCC	0.9285	1
**714**	TRANSURETHRAL PROSTATECTOMY WITHOUT CC/MCC	0.9285	1
**715**	OTHER MALE REPRODUCTIVE SYSTEM O.R. PROCEDURES FOR MALIGNANCY WITH CC/MCC	1.2765	2
**716**	OTHER MALE REPRODUCTIVE SYSTEM O.R. PROCEDURES FOR MALIGNANCY WITHOUT CC/MCC	1.2765	2
**717**	OTHER MALE REPRODUCTIVE SYSTEM O.R. PROCEDURES EXCEPT MALIGNANCY WITH CC/MCC	1.2362	2

© 2025 Mehtsun WT et al. *JAMA Health Forum*.

**718**	OTHER MALE REPRODUCTIVE SYSTEM O.R. PROCEDURES EXCEPT MALIGNANCY WITHOUT CC/MCC	1.2362	2
**734**	PELVIC EVISCERATION, RADICAL HYSTERECTOMY AND RADICAL VULVECTOMY WITH CC/MCC	1.4135	2
**735**	PELVIC EVISCERATION, RADICAL HYSTERECTOMY AND RADICAL VULVECTOMY WITHOUT CC/MCC	1.4135	2
**736**	UTERINE AND ADNEXA PROCEDURES FOR OVARIAN OR ADNEXAL MALIGNANCY WITH MCC	1.4766	3
**737**	UTERINE AND ADNEXA PROCEDURES FOR OVARIAN OR ADNEXAL MALIGNANCY WITH CC	1.4766	3
**738**	UTERINE AND ADNEXA PROCEDURES FOR OVARIAN OR ADNEXAL MALIGNANCY WITHOUT CC/MCC	1.4766	3
**739**	UTERINE AND ADNEXA PROCEDURES FOR NON-OVARIAN AND NON-ADNEXAL MALIGNANCY WITH MCC	1.2795	2
**740**	UTERINE AND ADNEXA PROCEDURES FOR NON-OVARIAN AND NON-ADNEXAL MALIGNANCY WITH CC	1.2795	2
**741**	UTERINE AND ADNEXA PROCEDURES FOR NON-OVARIAN AND NON-ADNEXAL MALIGNANCY WITHOUT CC/MCC	1.2795	2
**742**	UTERINE AND ADNEXA PROCEDURES FOR NON-MALIGNANCY WITH CC/MCC	1.1323	1
**743**	UTERINE AND ADNEXA PROCEDURES FOR NON-MALIGNANCY WITHOUT CC/MCC	1.1323	1
**744**	D&C, CONIZATION, LAPAROSCOPY AND TUBAL INTERRUPTION WITH CC/MCC	1.1705	1
**745**	D&C, CONIZATION, LAPAROSCOPY AND TUBAL INTERRUPTION WITHOUT CC/MCC	1.1705	1
**746**	VAGINA, CERVIX AND VULVA PROCEDURES WITH CC/MCC	0.938	1
**747**	VAGINA, CERVIX AND VULVA PROCEDURES WITHOUT CC/MCC	0.938	1
**748**	FEMALE REPRODUCTIVE SYSTEM RECONSTRUCTIVE PROCEDURES	1.3483	2
**749**	OTHER FEMALE REPRODUCTIVE SYSTEM O.R. PROCEDURES WITH CC/MCC	1.4659	3
**750**	OTHER FEMALE REPRODUCTIVE SYSTEM O.R. PROCEDURES WITHOUT CC/MCC	1.4659	3
**769**	POSTPARTUM AND POST ABORTION DIAGNOSES WITH O.R. PROCEDURES	1.6204	3
**770**	ABORTION WITH D&C, ASPIRATION CURETTAGE OR HYSTEROTOMY	0.8881	1
**783**	CESAREAN SECTION WITH STERILIZATION WITH MCC	0.9153	1
**784**	CESAREAN SECTION WITH STERILIZATION WITH CC	0.9153	1
**785**	CESAREAN SECTION WITH STERILIZATION WITHOUT CC/MCC	0.9153	1
**786**	CESAREAN SECTION WITHOUT STERILIZATION WITH MCC	0.8871	1
**787**	CESAREAN SECTION WITHOUT STERILIZATION WITH CC	0.8871	1
**788**	CESAREAN SECTION WITHOUT STERILIZATION WITHOUT CC/MCC	0.8871	1
**799**	SPLENECTOMY WITH MCC	1.6835	4
**800**	SPLENECTOMY WITH CC	1.6835	4
**801**	SPLENECTOMY WITHOUT CC/MCC	1.6835	4
**802**	OTHER O.R. PROCEDURES OF THE BLOOD AND BLOOD FORMING ORGANS WITH MCC	1.3642	2
**803**	OTHER O.R. PROCEDURES OF THE BLOOD AND BLOOD FORMING ORGANS WITH CC	1.3642	2
**804**	OTHER O.R. PROCEDURES OF THE BLOOD AND BLOOD FORMING ORGANS WITHOUT CC/MCC	1.3642	2
**817**	OTHER ANTEPARTUM DIAGNOSES WITH O.R. PROCEDURES WITH MCC	0.9979	1
**818**	OTHER ANTEPARTUM DIAGNOSES WITH O.R. PROCEDURES WITH CC	0.9979	1

© 2025 Mehtsun WT et al. *JAMA Health Forum*.

**819**	OTHER ANTEPARTUM DIAGNOSES WITH O.R. PROCEDURES WITHOUT CC/MCC	0.9979	1
**820**	LYMPHOMA AND LEUKEMIA WITH MAJOR O.R. PROCEDURES WITH MCC	1.2516	2
**821**	LYMPHOMA AND LEUKEMIA WITH MAJOR O.R. PROCEDURES WITH CC	1.2516	2
**822**	LYMPHOMA AND LEUKEMIA WITH MAJOR O.R. PROCEDURES WITHOUT CC/MCC	1.2516	2
**826**	MYELOPROLIFERATIVE DISORDERS OR POORLY DIFFERENTIATED NEOPLASMS WITH MAJOR O.R. PROCEDURES WITH MCC	1.6777	4
**827**	MYELOPROLIFERATIVE DISORDERS OR POORLY DIFFERENTIATED NEOPLASMS WITH MAJOR O.R. PROCEDURES WITH CC	1.6777	4
**828**	MYELOPROLIFERATIVE DISORDERS OR POORLY DIFFERENTIATED NEOPLASMS WITH MAJOR O.R. PROCEDURES WITHOUT CC/MCC	1.6777	4
**853**	INFECTIOUS AND PARASITIC DISEASES WITH O.R. PROCEDURES WITH MCC	1.495	3
**854**	INFECTIOUS AND PARASITIC DISEASES WITH O.R. PROCEDURES WITH CC	1.495	3
**855**	INFECTIOUS AND PARASITIC DISEASES WITH O.R. PROCEDURES WITHOUT CC/MCC	1.495	3
**856**	POSTOPERATIVE OR POST-TRAUMATIC INFECTIONS WITH O.R. PROCEDURES WITH MCC	1.3484	2
**857**	POSTOPERATIVE OR POST-TRAUMATIC INFECTIONS WITH O.R. PROCEDURES WITH CC	1.3484	2
**858**	POSTOPERATIVE OR POST-TRAUMATIC INFECTIONS WITH O.R. PROCEDURES WITHOUT CC/MCC	1.3484	2
**901**	WOUND DEBRIDEMENTS FOR INJURIES WITH MCC	1.1344	1
**902**	WOUND DEBRIDEMENTS FOR INJURIES WITH CC	1.1344	1
**903**	WOUND DEBRIDEMENTS FOR INJURIES WITHOUT CC/MCC	1.1344	1
**904**	SKIN GRAFTS FOR INJURIES WITH CC/MCC	1.6466	3
**905**	SKIN GRAFTS FOR INJURIES WITHOUT CC/MCC	1.6466	3
**906**	HAND PROCEDURES FOR INJURIES	1.7938	4
**907**	OTHER O.R. PROCEDURES FOR INJURIES WITH MCC	1.3746	2
**908**	OTHER O.R. PROCEDURES FOR INJURIES WITH CC	1.3746	2
**909**	OTHER O.R. PROCEDURES FOR INJURIES WITHOUT CC/MCC	1.3746	2
**927**	EXTENSIVE BURNS OR FULL THICKNESS BURNS WITH MV >96 HOURS WITH SKIN GRAFT	21.0309	5
**928**	FULL THICKNESS BURN WITH SKIN GRAFT OR INHALATION INJURY WITH CC/MCC	3.0089	5
**929**	FULL THICKNESS BURN WITH SKIN GRAFT OR INHALATION INJURY WITHOUT CC/MCC	3.0089	5
**955**	CRANIOTOMY FOR MULTIPLE SIGNIFICANT TRAUMA	6.288	5
**956**	LIMB REATTACHMENT, HIP AND FEMUR PROCEDURES FOR MULTIPLE SIGNIFICANT TRAUMA	3.8488	5
**957**	OTHER O.R. PROCEDURES FOR MULTIPLE SIGNIFICANT TRAUMA WITH MCC	2.7342	5
**958**	OTHER O.R. PROCEDURES FOR MULTIPLE SIGNIFICANT TRAUMA WITH CC	2.7342	5
**959**	OTHER O.R. PROCEDURES FOR MULTIPLE SIGNIFICANT TRAUMA WITHOUT CC/MCC	2.7342	5
**969**	HIV WITH EXTENSIVE O.R. PROCEDURES WITH MCC	2.9451	5
**970**	HIV WITH EXTENSIVE O.R. PROCEDURES WITHOUT MCC	2.9451	5
**981**	EXTENSIVE O.R. PROCEDURES UNRELATED TO PRINCIPAL DIAGNOSIS WITH MCC	1.6548	4

© 2025 Mehtsun WT et al. *JAMA Health Forum*.

**982**	EXTENSIVE O.R. PROCEDURES UNRELATED TO PRINCIPAL DIAGNOSIS WITH CC	1.6548	4
**983**	EXTENSIVE O.R. PROCEDURES UNRELATED TO PRINCIPAL DIAGNOSIS WITHOUT CC/MCC	1.6548	4

Source:
Centers for Medicare and Medicaid Services. FY 2021 Final Rule Tables and Correction Notice Tables - Table 5 (FY 2021 Final Rule and Correction Notice MS-DRGs, Relative Weighting Factors and Geometric and Arithmetic Mean Length of Stay). https://www.cms.gov/medicare/payment/prospective-payment-systems/acute-inpatient-pps/fy-2021-ipps-final-rule-home-page#Tables

Notes:
Inpatient surgical care episodes were identified using Diagnosis Related Group (DRG) codes, which were classified into medical and procedural categories by CMS. We identified DRGs specific to surgical episodes by excluding non-surgical procedures from the procedural DRGs.

© 2025 Mehtsun WT et al. *JAMA Health Forum*.

eFigure 1. Proportion of VA-Enrollees in HVMA Plans vs. Proportion of VA-Enrollees Among All Medicare Beneficiaries in Plan Service Area, 2021

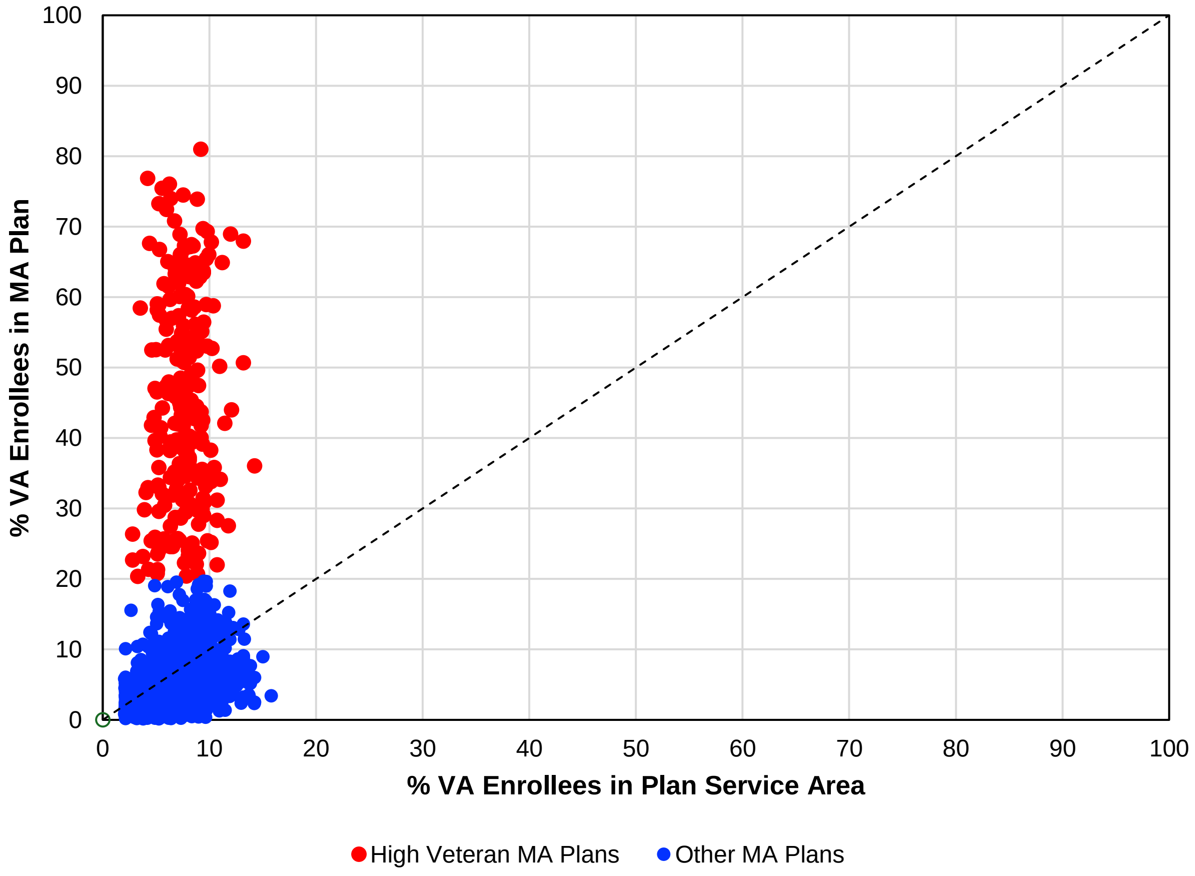

Notes:

a. Each dot represents an MA plan. The horizontal axis represents the proportion of VA-enrollees out of all Medicare beneficiaries in the plan's service area. The vertical axis represents the proportion of VA-enrollees in the MA plan.
b. High-veteran MA plans are defined as MA plans that enroll veterans exceeding 20 percent of their total enrollment in a given year. Employer-direct private fee-for-service plans, cost plans, Medicare Savings Account plans, and plans with less than 100 enrollees were excluded from the analyses.

© 2025 Mehtsun WT et al. *JAMA Health Forum*.

eFigure 2. Unadjusted Comparison of Inpatient Surgery Payment Sources for VA Enrollees in HVMA Plans and Other MA Plans, 2021

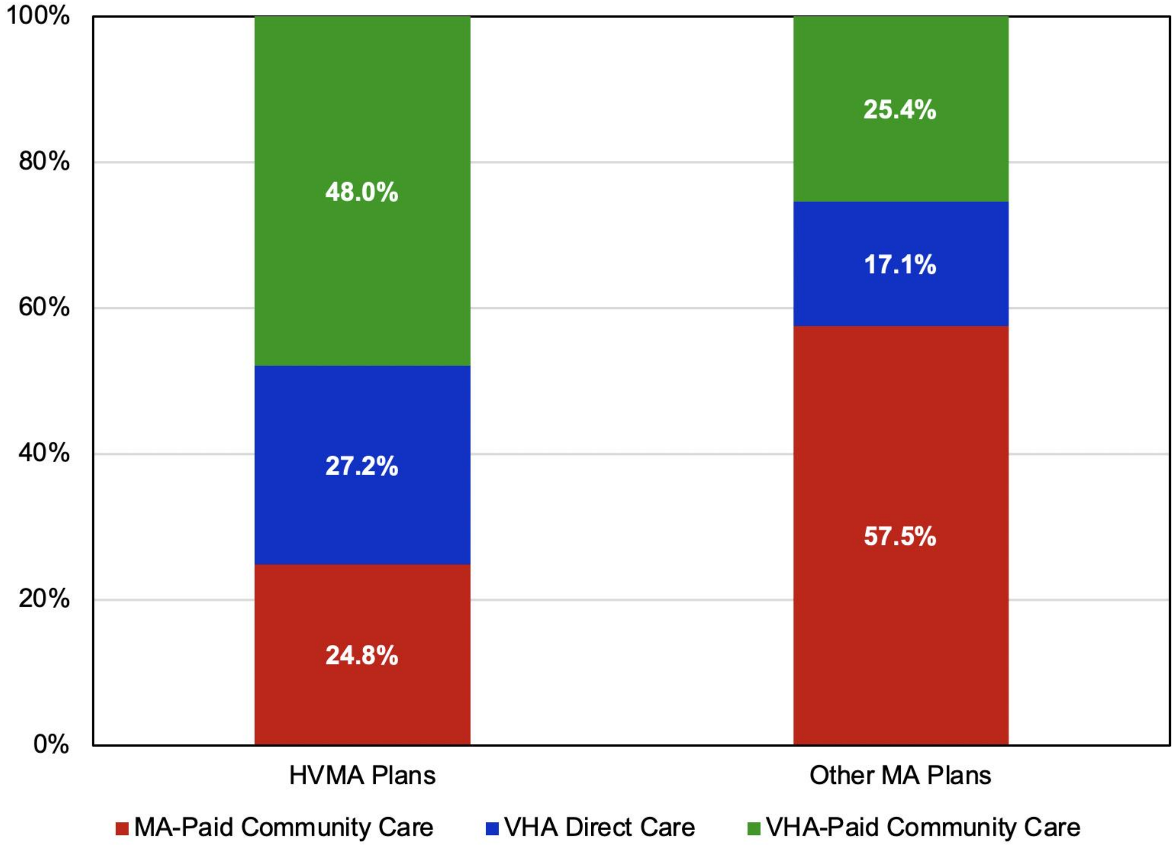

Notes:

1. High-veteran MA (HVMA) plans are defined as MA plans that enroll VA enrollees exceeding 20 percent of their total enrollment in 2021. Employer-direct private fee-for-service plans, cost plans, Medicare Savings Account plans, and plans with fewer than 100 enrollees were excluded from the analyses.
2. Unadjusted share represents the proportion of surgical episodes that are covered by MA plans, VA-paid community care, and VA-paid direct care respectively.

© 2025 Mehtsun WT et al. *JAMA Health Forum*.

eTable 3. Adjusted Comparison of Inpatient Surgery Payment Sources for VA Enrollees in HVMA Plans and Other MA Plans, 2021

	HVMA Plans	Other MA Plans	Difference	95% CI
MA-Paid Community Care				
**Overall**	30.5%	56.2%	-25.7%	(-26.7%,-24.6%)
**By Surgical Complexity**				
Q1 (Least Complex)	25.6%	53.5%	-27.9%	(-31.2%,-24.6%)
Q2	27.9%	55.1%	-27.2%	(-30.2%,-24.2%)
Q3	34.7%	58.6%	-23.9%	(-26.6%,-21.1%)
Q4	28.8%	55.8%	-27.0%	(-28.9%,-25.2%)
Q5 (Most Complex)	33.0%	56.6%	-23.6%	(-25.7%,-21.5%)
**By Source of Admission**				
Non-Elective	38.3%	61.1%	-22.8%	(-24.5%,-21.0%)
Elective	25.2%	52.5%	-27.3%	(-28.6%,-26.0%)
VA-Paid Community Care				
**Overall**	41.3%	26.6%	14.7%	(13.6%,15.8%)
**By Surgical Complexity**				
Q1 (Least Complex)	34.0%	21.9%	12.1%	(8.7%,15.4%)
Q2	40.4%	25.7%	14.6%	(11.6%,17.7%)
Q3	41.4%	26.8%	14.6%	(11.9%,17.3%)
Q4	36.6%	23.6%	13.0%	(11.1%,14.8%)
Q5 (Most Complex)	50.1%	32.3%	17.8%	(15.7%,20.0%)
**By Source of Admission**				
Non-Elective	42.2%	26.6%	15.7%	(13.9%,17.4%)
Elective	40.7%	26.6%	14.1%	(12.7%,15.5%)
VA-Paid Direct Care				
**Overall**	28.3%	17.2%	11.0%	(10.0%,12.0%)
**By Surgical Complexity**				
Q1 (Least Complex)	40.4%	24.6%	15.8%	(12.3%,19.3%)
Q2	31.8%	19.2%	12.6%	(9.6%,15.6%)
Q3	23.9%	14.6%	9.2%	(6.9%,11.6%)
Q4	34.6%	20.5%	14.1%	(12.2%,15.9%)
Q5 (Most Complex)	16.9%	11.1%	5.8%	(4.1%,7.4%)
**By Source of Admission**				
Non-Elective	19.4%	12.3%	7.1%	(5.7%,8.5%)
Elective	34.0%	20.8%	13.2%	(11.9%,14.6%)

Notes:

1. High-veteran MA (HVMA) plans are defined as MA plans that enroll VA enrollees exceeding 20 percent of their total enrollment in 2021. Employer-direct private fee-for-service plans, cost plans, Medicare Savings Account plans, and plans with fewer than 100 enrollees were excluded from the analyses.

© 2025 Mehtsun WT et al. *JAMA Health Forum*.

2. Multinomial logistic regression models were estimated to evaluate the association between veteran enrollment in HVMA plans and the likelihood of their surgical care being paid by each of the three payment sources. Separate regression models were estimated for each surgical complexity quintile and each source of admission. The dependent variable is the payment source of the surgical episode, and the key explanatory variable is whether the VA enrollee is enrolled in a HVMA plan. Adjusted difference represents the difference in the proportion of surgical episodes that are covered by MA plans, VA-paid community care, and VA-paid direct care respectively for VA enrollees in high-veteran MA (HVMA) plans relative to other MA plans. Analyses adjusted for VA enrollees' age, gender, race and ethnicity, Medicare and Medicaid dual eligibility, rurality, priority group designations, surgical complexity, and state fixed effects.

© 2025 Mehtsun WT et al. *JAMA Health Forum*.

eTable 4. Adjusted Comparison of Inpatient Surgery Payment Sources for VA Enrollees in HVMA Plans and Other MA Plans Using Logistic Regressions, 2021

	HVMA Plans	Other MA Plans	Difference	95% CI
VA-Paid Care				
**Overall**	69.5%	43.8%	25.7%	(24.6%, 26.7%)
**By Surgical Complexity**				
Q1 (Least Complex)	74.4%	46.6%	27.8%	(24.5%, 31.2%)
Q2	72.1%	44.9%	27.2%	(24.2%, 30.2%)
Q3	65.3%	41.4%	23.9%	(21.2%, 26.6%)
Q4	71.1%	44.2%	27.0%	(25.1%, 28.8%)
Q5 (Most Complex)	67.1%	43.4%	23.7%	(21.6%, 25.7%)
**By Source of Admission**				
Non-Elective	61.7%	38.9%	22.8%	(21.0%, 24.5%)
Elective	74.8%	47.4%	27.3%	(26.0%, 28.6%)
VA-Paid Community Care				
**Overall**	59.7%	61.0%	-1.3%	(-2.6%, 0.1%)
**By Surgical Complexity**				
Q1 (Least Complex)	45.2%	47.4%	-2.2%	(-6.5%, 2.1%)
Q2	56.4%	57.4%	-1.1%	(-4.9%, 2.8%)
Q3	63.1%	64.7%	-1.6%	(-5.0%, 1.9%)
Q4	51.4%	53.9%	-2.6%	(-4.9%, -0.2%)
Q5 (Most Complex)	75.3%	74.3%	1.0%	(-1.4%, 3.4%)
**By Source of Admission**				
Non-Elective	68.2%	68.4%	-0.3%	(-2.4%, 1.9%)
Elective	54.9%	56.3%	-1.4%	(-3.1%, 0.3%)

Notes:

1. High-veteran MA (HVMA) plans are defined as MA plans that enroll VA enrollees exceeding 20 percent of their total enrollment in 2021. Employer-direct private fee-for-service plans, cost plans, Medicare Savings Account plans, and plans with fewer than 100 enrollees were excluded from the analyses.
2. A two-part logistic regression model was estimated to assess the association between veteran enrollment in HVMA plans and the payment source of their surgical care. In the first part of the two-part model, we estimated the association between veteran enrollment in HVMA plans and the likelihood of their surgical care being paid by the VA rather than the MA plans using a logistic regression model where the dependent variable is a binary indicator of whether a VA enrollee's surgical care is paid by the VA. In the second part of the model, we estimated the association between veteran enrollment in HVMA plans and the likelihood

© 2025 Mehtsun WT et al. *JAMA Health Forum.*

of receiving VA-paid community care conditional on the VA enrollee's surgical care was paid by the VA using a logistic regression model where the dependent variable is a binary indicator of whether a VA enrollee's surgical care is received through VA-paid community care. Both regressions adjusted for VA enrollees' age, gender, race and ethnicity, Medicare and Medicaid dual eligibility, rurality, priority group designations, surgical complexity, and state fixed effects.

© 2025 Mehtsun WT et al. *JAMA Health Forum*.

eTable 5. Adjusted Comparison of Inpatient Surgery Payment Sources for VA Enrollees in HVMA Plans and Other MA Plans, Plans with High Data Completeness Only, 2021

	HVMA Plans	Other MA Plans	Difference	95% CI
MA-Paid Community Care				
**Overall**	30.4%	56.9%	-26.5%	(-27.6%,-25.4%)
**By Surgical Complexity**				
Q1 (Least Complex)	25.5%	54.5%	-29.0%	(-32.3%,-25.6%)
Q2	27.6%	56.0%	-28.4%	(-31.4%,-25.3%)
Q3	35.5%	58.9%	-23.5%	(-26.2%,-20.7%)
Q4	28.4%	56.6%	-28.1%	(-30.0%,-26.3%)
Q5 (Most Complex)	33.0%	57.2%	-24.3%	(-26.4%,-22.2%)
**By Source of Admission**				
Non-Elective	38.1%	61.8%	-23.7%	(-25.5%,-21.9%)
Elective	25.2%	53.2%	-28.0%	(-29.3%,-26.6%)
VA-Paid Community Care				
**Overall**	41.4%	26.2%	15.2%	(14.1%,16.3%)
**By Surgical Complexity**				
Q1 (Least Complex)	34.4%	21.1%	13.3%	(9.9%,16.7%)
Q2	40.8%	25.4%	15.4%	(12.2%,18.5%)
Q3	40.8%	26.6%	14.2%	(11.5%,16.9%)
Q4	37.1%	23.2%	13.8%	(12.0%,15.7%)
Q5 (Most Complex)	50.1%	31.9%	18.3%	(16.1%,20.4%)
**By Source of Admission**				
Non-Elective	43.0%	26.1%	16.9%	(15.1%,18.7%)
Elective	40.5%	26.3%	14.2%	(12.8%,15.6%)
VA-Paid Direct Care				
**Overall**	28.2%	16.9%	11.3%	(10.2%,12.3%)
**By Surgical Complexity**				
Q1 (Least Complex)	40.1%	24.5%	15.6%	(12.1%,19.2%)
Q2	31.6%	18.6%	13.0%	(10.0%,16.0%)
Q3	23.7%	14.5%	9.3%	(6.9%,11.7%)
Q4	34.5%	20.2%	14.3%	(12.4%,16.2%)
Q5 (Most Complex)	16.9%	10.9%	6.0%	(4.3%,7.7%)
**By Source of Admission**				
Non-Elective	18.9%	12.1%	6.8%	(5.4%,8.2%)
Elective	34.3%	20.6%	13.7%	(12.4%,15.1%)

© 2025 Mehtsun WT et al. *JAMA Health Forum*.

Notes:

1. High-veteran MA (HVMA) plans are defined as MA plans that enroll VA enrollees exceeding 20 percent of their total enrollment in 2021. Employer-direct private fee-for-service plans, cost plans, Medicare Savings Account plans, and plans with fewer than 100 enrollees were excluded from the analyses.
2. Multinomial logistic regression models were estimated to evaluate the association between veteran enrollment in HVMA plans and the likelihood of their surgical care being paid by each of the three payment sources. Separate regression models were estimated for each surgical complexity quintile and each Source of admission. The dependent variable is the payment source of the surgical episode, and the key explanatory variable is whether the VA enrollee is enrolled in a HVMA plan. Adjusted difference represents the difference in the proportion of surgical episodes that are covered by MA plans, VA-paid community care, and VA-paid direct care respectively for VA enrollees in high-veteran MA (HVMA) plans relative to other MA plans. Analyses adjusted for VA enrollees' age, gender, race and ethnicity, Medicare and Medicaid dual eligibility, rurality, priority group designations, surgical complexity, and state fixed effects.

© 2025 Mehtsun WT et al. *JAMA Health Forum*.
