# Supplementary material for: Payment Source Shift for Surgical Care Among Veterans Enrolled in Medicare Advantage Plans
Source: JAMA Health Forum. 2025 Jun 9;6(6):e250827. doi: 10.1001/jamahealthforum.2025.0827 (PMC12150190; doi:10.1001/jamahealthforum.2025.0827)
Supplement: Supplement 2. — Data Sharing Statement [file jamahealthforum-e250827-s002.pdf]

Data Sharing Statement

Mehtsun. Payment Source Shift for Surgical Care Among Veterans Enrolled in Medicare Advantage Plans. *JAMA Health Forum*. Published June 09, 2025.
doi:10.1001/jamahealthforum.2025.0827

Data
Data available: No

Additional Information
Explanation for why data not available: These data cannot be shared due to data use agreement with the Department of Veterans Affairs.
